# Multi-omics subtyping of hepatocellular carcinoma patients using a Bayesian network mixture model

**DOI:** 10.1371/journal.pcbi.1009767

**Published:** 2022-09-06

**Authors:** Polina Suter, Eva Dazert, Jack Kuipers, Charlotte K. Y. Ng, Tuyana Boldanova, Michael N. Hall, Markus H. Heim, Niko Beerenwinkel

**Affiliations:** 1 Department of Biosystems Science and Engineering, ETH Zurich, Basel, Switzerland; 2 SIB Swiss Institute of Bioinformatics, Lausanne, Switzerland; 3 Biozentrum, University of Basel, Basel, Switzerland; 4 Department for BioMedical Research (DBMR), University of Bern, Bern, Switzerland; 5 Department of Biomedicine, University Hospital Basel, University of Basel, Basel, Switzerland; 6 Institute of Medical Genetics and Pathology, University Hospital Basel, University of Basel, Basel, Switzerland; 7 Department of Gastroenterology and Hepatology, Clarunis, University Center for Gastrointestinal and Liver Diseases, Basel, Switzerland; Heidelberg University, GERMANY

## Abstract

Comprehensive molecular characterization of cancer subtypes is essential for predicting clinical outcomes and searching for personalized treatments. We present bnClustOmics, a statistical model and computational tool for multi-omics unsupervised clustering, which serves a dual purpose: Clustering patient samples based on a Bayesian network mixture model and learning the networks of omics variables representing these clusters. The discovered networks encode interactions among all omics variables and provide a molecular characterization of each patient subgroup. We conducted simulation studies that demonstrated the advantages of our approach compared to other clustering methods in the case where the generative model is a mixture of Bayesian networks. We applied bnClustOmics to a hepatocellular carcinoma (HCC) dataset comprising genome (mutation and copy number), transcriptome, proteome, and phosphoproteome data. We identified three main HCC subtypes together with molecular characteristics, some of which are associated with survival even when adjusting for the clinical stage. Cluster-specific networks shed light on the links between genotypes and molecular phenotypes of samples within their respective clusters and suggest targets for personalized treatments.

## Author summary

Multi-omics approaches to cancer subtyping can provide more insights into molecular changes in tumors compared to single-omics approaches. However, most multi-omics clustering methods do not take into account that gene products interact, for example, as parts of protein complexes or signaling networks. Here we present bnClustOmics, a Bayesian network mixture model for unsupervised clustering of multi-omics data, which can represent dependencies among molecular changes of various omics types explicitly. Unlike other approaches that use data from public interaction databases as ground truth, bnClustOmics learns the dependencies between genes from the analyzed multi-omics dataset. At the same time, our approach can also account for prior knowledge from public interaction databases and use it to guide network learning without losing the ability to learn new dependencies. We applied bnClustOmics to a multi-omics HCC dataset and identified three subtypes similar to those identified in other HCC studies. The cluster-specific networks learned by bnClustOmics revealed additional insights into the molecular characterization of the discovered subgroups and highlighted the changes in signaling networks leading to distinct HCC phenotypes.

## Introduction

Cancer is a complex disease and one of the leading causes of death worldwide. Over the last decades, much research was devoted to discovering cancer subtypes based on genomic and transcriptomic data [1–3]. Molecular subtyping approaches based on gene expression have been helpful for the identification of markers associated with clinical outcomes and facilitated the search for targeted therapies [4, 5]. More recently, cancer subtyping has been based on integrating multiple different omics types [6–9]. Multiple tools have been developed to integrate multi-omics data and learn interaction networks to understand what drives oncogenesis [10, 11]. However, our understanding of how heterogeneous genetic alterations in cancer cells affect signaling pathways and lead to a few disease phenotypes is still far from complete [12, 13]. One major obstacle is the missing connection between methods for network discovery and approaches to molecular subtyping. Almost all existing methods focus on only one of these two tasks.

Only a few multi-omics clustering methods include interactions between gene products into the model explicitly. Some of them are designed for single omics types [14–16] or use a supervised approach for clustering [17]. PARADIGM [18] is the only tool that performs unsupervised clustering (of patient samples) while accounting for the fact that gene products can interact with each other and that interactions may differ between patient groups. However, this method relies entirely on existing protein-protein interaction (PPI) databases and considers them as ground truth. Instead of learning the network from the dataset, PARADIGM maps the omics data onto interactions from existing databases by considering pairwise directed dependencies between genes. Hence this tool is prone to mistakes contained in the curated databases and does not allow the discovery of unknown interactions. This shortcoming is exacerbated by the fact that PARADIGM requires a very detailed prior where all interactions must be directed and biologically defined. Hence many interaction databases cannot be incorporated into such a prior. Finally, this tool cannot be applied to all omics types. For example, it cannot be applied to phosphoproteomics data that contains multiple phosphorylation sites of the same gene.

When learning gene regulatory networks, the Bayesian network framework is often used instead of pairwise correlation analysis since it can uncover direct interactions and, in some cases, learn their directions [19, 20]. A Bayesian network mixture model was used for clustering of pan-cancer mutation data [14], but never applied to any other omics types or integrated multi-omics model for unsupervised clustering.

Here, we extend the model of Kuipers et al. [14] to multi-omics data comprising discrete and continuous data types. We present bnClustOmics, an unsupervised clustering method based on the assumption that the cancer subtype can be represented as a Bayesian network consisting of omics variables of various types. Our model reflects the consensus view of cancer mechanisms, in which genetic alterations disrupt normal cell signaling and activate oncogenic pathways. Biological experiments have shown that mutations in cancer cells result in altered interactions between proteins, including phosphoproteins [21]. Thus, modeling the subtype-specific changes in the interactome may improve the clustering model. With cancer subtypes being modeled as Bayesian networks, bnClustOmics can detect the signal from interactions that differ in networks representing different subtypes. A major advantage of bnClustOmics compared to other methods for multi-omics clustering is that the output includes networks (learned *de novo*) representing discovered clusters which can be considered further in downstream analyses and shed light on subtype-specific cancer mechanisms.

We demonstrated in simulation studies that many commonly used clustering methods, including those specifically designed for multi-omics data, have a limited ability to detect a signal from changed interactions, whereas the ability of bnClustOmics to do so improves its clustering accuracy. In particular, we compared traditional clustering approaches with three different approaches designed for multi-omics data. Among multi-omics approaches, we selected methods that demonstrated good performance in previous benchmarking studies [6, 22, 23]. iClusterPlus [24] uses a regularized latent variable model and provides a tool to tune the sparsity parameter. CIMLR [25] builds a similarity matrix based on multiple Gaussian kernels per data type and can incorporate the complete genome without enforcing sparsity. CIMLR was expected to perform better in a broad range of settings due to its claimed ability to learn the importance of different omics types from the analyzed dataset [25]. We also added MOFA [26] to benchmarking since it demonstrated good results with regard to feature selection in our simulations. bnClustOmics is only feasible for a limited number of omics features, hence the importance of each omics type is implicitly affected by the feature selection method. We tried several approaches to select relevant features and compared the performance of bnClustOmics using a selected subset to all other clustering methods applied to a non-reduced set.

We applied bnClustOmics to a multi-omics dataset from hepatocellular carcinoma (HCC) patient biopsies [27]. HCC is the most common type of primary liver cancer, which is the fourth most common cause of cancer-related mortality worldwide [28]. We discovered three clusters of HCC patients based on five omics types: mutations and copy number changes (both genome), transcriptome, proteome, and phosphoproteome. The number and molecular characteristics of the three discovered groups confirm many findings from previous HCC studies, including an analysis of the same HCC dataset [27]. In addition to cluster assignments, we analyzed the cluster-specific networks learned by bnClustOmics and scrutinized specific edges which connect changes in the genome to abnormal expression of transcripts, proteins, and phosphorylation sites. Furthermore, we identified hub nodes, i.e. genes with the most stable and most varying neighbors across cluster-specific networks based on the posterior probabilities of the edges. Cluster-specific connections between omics variables provide insights into the molecular characteristics underlying HCC subtypes and suggest targets for personalized therapies.

## 1 Results and discussion

### 1.1 Model and workflow

We model a cancer subtype as a Bayesian network, whose nodes represent different omics measurements of the same set of genes. The HCC dataset [27] includes five omics types, namely mutations and copy number changes (both genome, denoted *M* and *CN*), transcriptome (*T*), proteome (*P*), and phosphoproteome (*PP*). The edges in the network represent statistical dependencies among all observations across all omics types. Such dependencies are not limited to a single biological interpretation. For example, an edge in the network might represent a physical interaction between proteins, a regulatory relationship between a transcription factor and its target, a functional interaction or a co-expression pattern. A functional interaction denotes an indirect association where two gene products do not physically interact but are jointly involved in the same cellular process [29].

By design, our integrative model prohibits any edges from nodes of continuous data types (*T*, *P*, *PP*) to nodes of binary or ordinal data types (*M*, *CN*). Prohibiting interactions between certain omics types avoids overfitting and results in more interpretable networks. We only allow edges aligned with the information flow of the central dogma of molecular biology [30].

**Fig 1:**
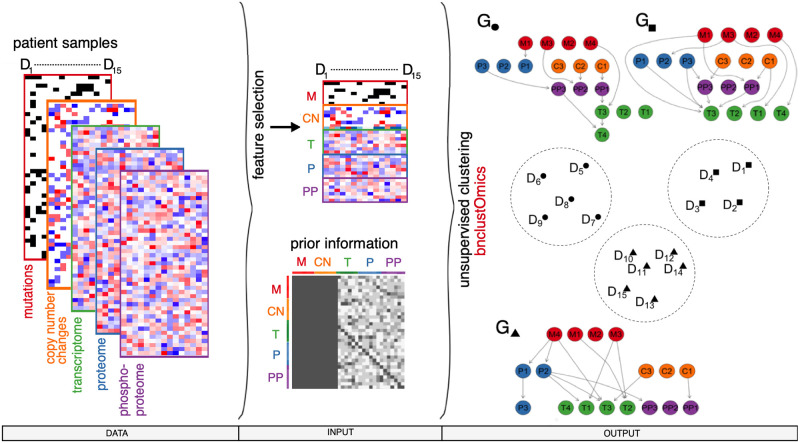
Bayesian network-based clustering workflow. Multiple omics types, both binary and continuous, are allowed as input data types (left). After feature selection is performed, prior knowledge about interactions between nodes can be included via blacklisting and penalization matrices (middle). bnClustOmics performs unsupervised clustering based on the selected features, blacklist, and penalization matrices. The output (right) includes cluster assignments (encircled patient sample), cluster-specific networks, and posterior probabilities of all individual edges in these graphs. Here, three patient clusters are depicted and labeled ●, ▲, and ■.

At the first step of the analysis, we perform feature selection from all features of all available omics types (Fig 1). To analyze the HCC dataset, we selected features based on multi-omics factor analysis (MOFA, [26]) latent factor analysis, differential gene expression (DGE) analysis, and prior knowledge about signaling networks (Section 2.13).

bnClustOmics uses a Bayesian network mixture model and employs the EM algorithm [14] to cluster patient samples and learn the networks representing those clusters. Unlike other multi-omics clustering methods, bnClustOmics does not rely on interactions from databases, but learns Bayesian networks from data *de novo* using a Bayesian approach [14, 31, 32]. However, it is possible to construct blacklist and penalization matrices that incorporate prior information about interactions between selected features and guide network learning in subsequent steps. Blacklisted interactions cannot be discovered at the network learning step. Thus, blacklisting requires a high degree of confidence that the interaction does not occur in any biological context. For this reason, blacklisting all edges which are not found in a specific database, is not recommended. Instead, we can use edge-specific penalization factors to modify the prior probability distribution of the graph structure and lower the probability of such edges appearing in the resulting graphs. The penalization matrix also provides an easy way to incorporate a confidence score which is often assigned to interactions in the PPI databases. Blacklisting and penalization matrices may have a substantial impact on the discovered networks, while their effect on clustering is generally small because the graphical prior is common for all clusters in an unsupervised setting.

bnClustOmics takes as input the observed values of the selected omics features for all patients, the number of clusters, and optional blacklist and penalization matrices. As output, we obtain cluster assignments for all patient samples, cluster-specific networks consisting of omics variables, and the log-likelihood, AIC and BIC scores of the estimated model. The AIC and BIC can be used to determine the optimal number of clusters. In addition, the Bayesian method used for structure learning provides estimates of posterior probabilities of all edges in the discovered networks. The statistical model presented in this work is implemented in the R package bnClustOmics and available at the GitHub repository https://github.com/cbg-ethz/bnClustOmics.

### 1.2 Benchmarking

We compared the performance of several clustering algorithms to bnClustOmics when the data generating model is a mixture of Bayesian networks. For comparison, we selected several general clustering methods as well as methods specifically designed for integration and clustering of multi-omics data, including kmeans [33], hclust [33], mclust [34], iClusterPlus [24], CIMLR [25], and MOFA [26].

For each set of simulation settings, we generated 30–50 Bayesian network mixtures (Section 2.2), where each directed acyclic graph (DAG) *G*_*k*_ consists of *n*_*c*_ Gaussian and *n*_*b*_ Bernoulli random variables (S1 Appendix). We used the adjusted Rand index (ARI, [35]), precision and F1 measures to measure the accuracy of clustering.

**Fig 2:**
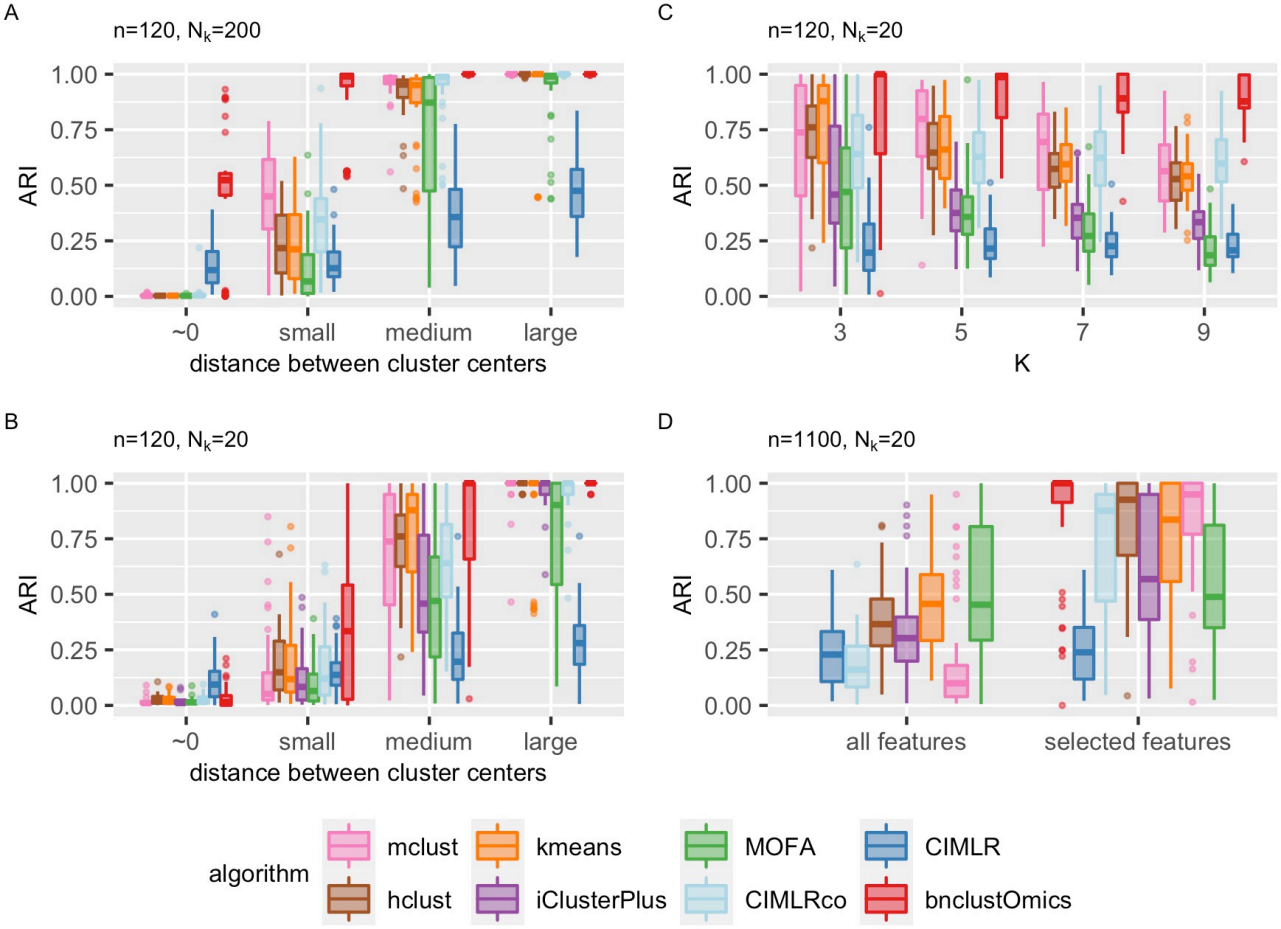
Benchmarking of algorithms for unsupervised clustering of multi-omics data. 50 Bayesian network mixtures were generated for each simulation setting. For general clustering approaches, the dimension was reduced by applying PCA and running clustering on the first 5 principal components. All integrative multi-omics approaches were applied to the original data unless specified otherwise. CIMLRco denotes clustering results of the application of CIMLR to a subset of data consisting of observations of only continuous variables. $N_{Z_{k}}$ denotes the number of observations in one cluster, *K* the number of clusters, *n*_*c*_ number of continuous nodes, *n*_*b*_ number of binary nodes in networks. (A) *K* = 3, *n*_*c*_ = 100, *n*_*b*_ = 20, $N_{Z_{k}}=200$ (B) *K* = 3, *n*_*c*_ = 100, *n*_*b*_ = 20, $N_{Z_{k}}=20$ (C) *n*_*c*_ = 100, *n*_*b*_ = 20, $N_{Z_{k}}=20$, *K* ∈ {3, 5, 7, 9}; distance between centers set to medium (D) *K* = 3, *n*_*c*_ = 1000, *n*_*b*_ = 100, $N_{Z_{k}}=20$, algorithms were applied to the full data and a subset of data consisting of all binary nodes with non-zero standard deviation and 150 selected continuous nodes; distance between centers set to medium.

For large sample sizes, bnClustOmics reaches a high accuracy even in a setting where the difference between cluster centers is small (Fig 2A, S1 Appendix), while the other algorithms fail to discover cluster assignments when cluster centers are very close to each other. Accuracy improves when the distances between centers of mixture components become larger for all algorithms except CIMLR. In our simulation settings, CIMLR failed to detect the signal from the continuous nodes in the presence of binary nodes. When we removed binary nodes from the simulated datasets and applied CIMLR to the continuous part only, its accuracy improved considerably.

For small sample sizes, all methods demonstrate lower clustering accuracy (Fig 2B, S1(B) and S2(B) Figs) and bnClustOmics outperforms the other approaches in the majority of cases. We attribute this outperformance to the ability of bnClustOmics to detect the signal not only from differences between cluster centers but also structural differences between graphs representing clusters.

Next, we fix the distance between the centers of the distributions at a medium value and analyze the performance of different algorithms with four different values *K* of the number of clusters. The clustering accuracy of bnClustOmics does not become worse with increasing number of clusters *K*, while for the other algorithms, the accuracy decreases (Fig 2C S1(C) and S2(C) Figs). Among the other algorithms, CIMLR applied to only a continuous part of the data performs the best. However, its accuracy is again substantially worse when the binary data is included.

Since bnClustOmics is only computationally feasible for networks with a limited number of nodes, its performance may strongly depend on the selection of the relevant features. To assess whether reducing the number of features affects the clustering accuracy of bnClustOmics, we generated Bayesian network mixtures with *n*_*c*_ = 1000 Gaussian nodes and *n*_*b*_ = 100 binary nodes from *K* = 3 mixture components. All algorithms were applied to the complete and reduced datasets, except bnClustOmics which was applied to only the reduced dataset. The reduced dataset included continuous features selected based on their weights in the latent factors identified by MOFA (S2 Appendix) and all binary features with at least one non-zero observation. Interestingly, all algorithms performed better using on the reduced dataset. However, bnClustOmics outperformed the other methods when the distance between cluster centers was medium (Fig 2D, S1(D) and S3(A)–S3(D) Figs).

Next, we tested the ability of bnClustOmics to reconstruct Bayesian networks representing discovered clusters. We generated 50 Bayesian network mixtures with *K* = 4 components and unequal weights, such that the four clusters contain 150, 100, 50, and 20 observations, respectively. The Bayesian approach yields estimated *maximum a posteriori* (MAP) structures, i.e. graphs which have the highest scores of all considered structures and represent the best fit to the data. In addition to MAP graphs, we also estimated the consensus structures (Section 2.5), which consist of edges whose posterior probabilities are higher than a certain threshold.

**Fig 3:**
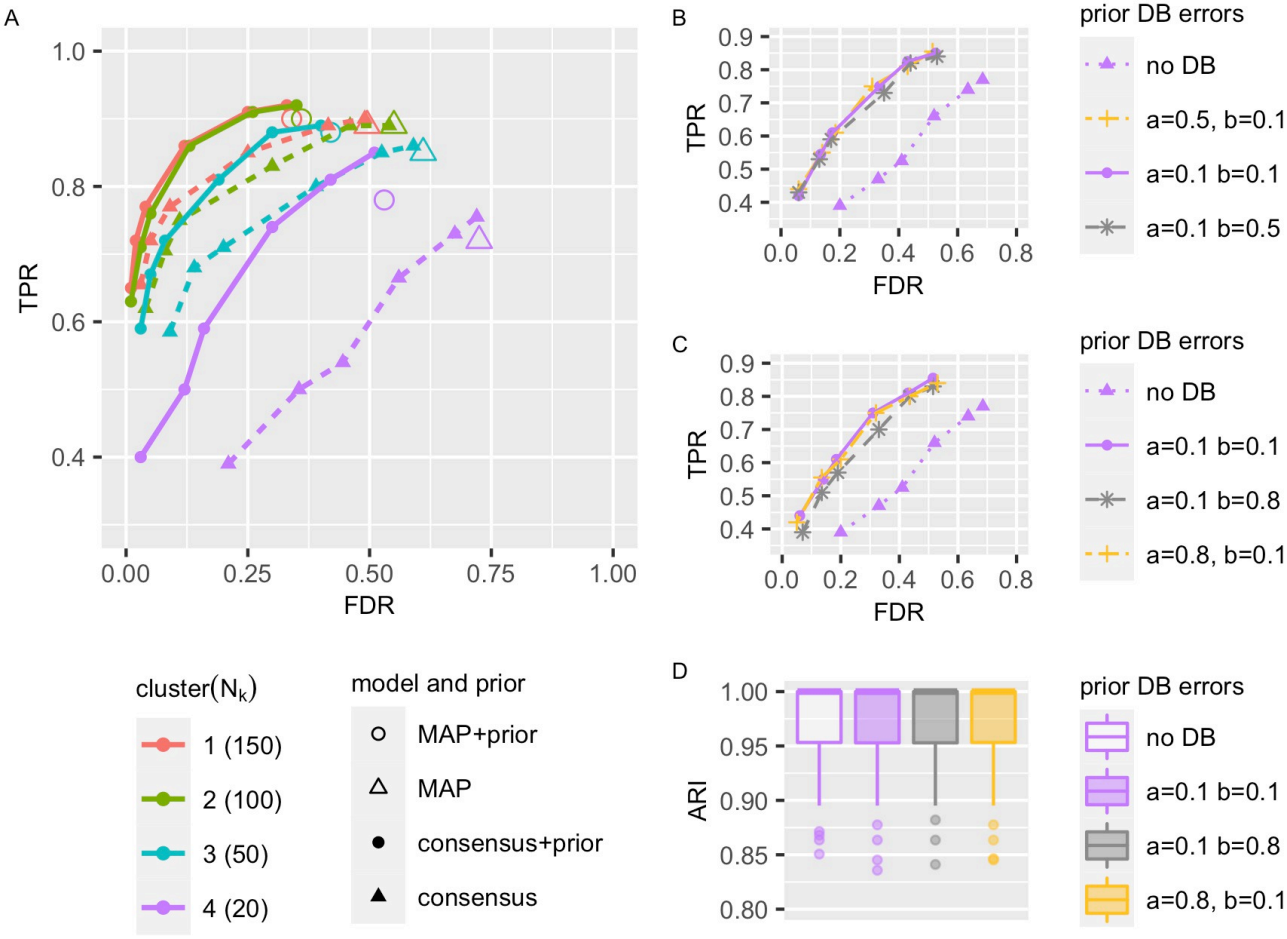
Structure fit. 50 datasets were generated from Bayesian network mixtures consisting of *K* = 4 components with number of observations $N_{Z_{k}}\in \left\{150,100,50,20\right\}$ corresponding to cluster 1 (red), cluster 2 (green), cluster 3 (turquoise) and cluster 4 (violet). To construct the penalization matrix (prior), we first defined the edges representing interactions from databases by taking the union of all edges in the ground truth structures. Afterward, we removed 10% of these edges, modeling false-negative interactions in databases (*b* = 0.1), and added 10% of false positives (*a* = 0.1). The entries of the penalization matrix corresponding to the defined set were not penalized; all other edges were penalized by a factor of two. The simulated datasets were clustered using bnClustOmics with and without the penalization matrix. Resulting MAP and consensus models corresponding to posterior thresholds of *p* ∈ {0.3, 0.5, 0.7, 0.9, 0.95, 0.99} were assessed using TPR and FDR. (B) Additional curves were added for cluster 4 visualizing results for simulated databases constructed using various levels of FDR(a) and FNR(b): *a* = 0.1, *b* = 0.1 (violet solid), *a* = 0.5, *b* = 0.1 (yellow) and *a* = 0.1, *b* = 0.5 (grey). (C) *a* = 0.8, *b* = 0.1 (yellow) and *a* = 0.1, *b* = 0.8 (grey). (D) Clustering accuracy: no database (white), *a* = 0.1, *b* = 0.1 (violet), *a* = 0.5, *b* = 0.1 (yellow), *a* = 0.1, *b* = 0.5 (grey).

The number of observations per cluster correlates positively with the accuracy of the learned MAP structures, as progressively higher TPR and lower FDR levels were reported for MAP structures corresponding to a higher number of observations (Fig 3A). However, the FDR of MAP structures is rather high, especially for the cluster 4 with the smallest number of observations. We observe that consensus graphs help reduce FDR compared to the MAP estimates, although at the cost of reducing the true positive rate (TPR). The structural Hamming distance (SHD) is smaller for consensus structures than for MAP structures (S5 Fig). In our simulation, stringent posterior thresholds of 0.9–0.95 minimize the SHD for all $N_{Z_{k}}$.

The Bayesian approach allows us to include prior knowledge about known interactions and guide *de novo* network learning. In the analysis of mutation data, an edge penalization matrix was used by Kuipers et al. [14] to include prior information from the database STRING [36]. The edge penalization matrix is used to modify the prior over structures, such that penalized edges have a lower chance to appear in the discovered graphs (Section 2.9). PPI databases contain known interactions between genes but most often do not describe the context in which a particular interaction occurs. Hence, if interactions differ between unknown cancer subtypes, we cannot learn them using a database alone. To assess to which extent the penalization matrix can improve network discovery, we constructed a simulated database of interactions by taking the union of all edges in the ground truth structures and introducing 10% of mistakes which model false-positive (10%) and false-negative (10%) interactions in databases. The entries of the penalization matrix corresponding to interactions from the simulated database were not penalized; all other edges were penalized by a factor of two.

The usage of an edge penalization matrix resulted in MAP and consensus structures containing fewer false-positive edges and more true positives than corresponding structures obtained without using a penalization matrix (Fig 3A). Limited sample size is a common problem of biological data, and proteome and phosphoproteome data are generally scarce. At the same time, extensive databases exist which include known protein-protein interactions and regulatory relationships identified in biological or computational studies. Hence, including information from such databases can be helpful for network reconstruction. However, the databases are known to be different concerning the quantity and quantity of included interactions. We compared the results of structure fit for the smallest cluster 4 where the prior provided the most considerable advantage for three simulated databases, obtained by varying parameters *a* and *b* representing the FDR and the FNR of the database, respectively. Strikingly, the structure fit remained equally good compared to using no prior, even in extreme cases of 80% FDR or 80% FNR (Fig 3B and 3C). The prior database lacking 80% of the true edges was only marginally worse than the others (Fig 3C). This happens because even when the database contains 80% of false-positive or lacks 80% or true-positive interactions, it still penalizes more than 90% of edges in the search space and provides enough guidance for the structure learning algorithm. Finally, the graphical prior did not affect the clustering accuracy (Fig 3D). This was expected since the prior is not cluster-specific in an unsupervised setting.

Finally, bnClustOmics allows estimating the number of clusters *K* using either the AIC or BIC score. Our simulations indicate that for small sample size, AIC works better (S4(A) Fig), while for large sample size, BIC shows better results (S4(B) Fig).

### 1.3 HCC patient subtyping

We analyzed the HCC multi-omics dataset [27] comprising 50 biopsies from 48 patients and including five omics types, namely mutations and CNAs (both genome), transcriptome, proteome, and phosphoproteome. In order to apply bnClustOmics, we first performed feature selection as follows. To select *M* features, we used a list of significantly mutated genes in the analyzed cohort identified by Ng et al. [27]. In addition, we included possible drivers of HCC identified in other studies [37–39]. To select continuous features, we applied MOFA and performed latent factor analysis. In addition, we included the *P* and *PP* features, which are differentially expressed/phosphorylated in tumor samples and either are present in the kinase-substrate database, or are known transcription factors according to the Omnipath database [40] (Section 2.10). We proceeded with the construction of the blacklist and penalization matrices as described in Section 2.8 and Section 2.9 and included prior information about interactions between selected features from the STRING and Omnipath databases [36, 40].

**Fig 4:**
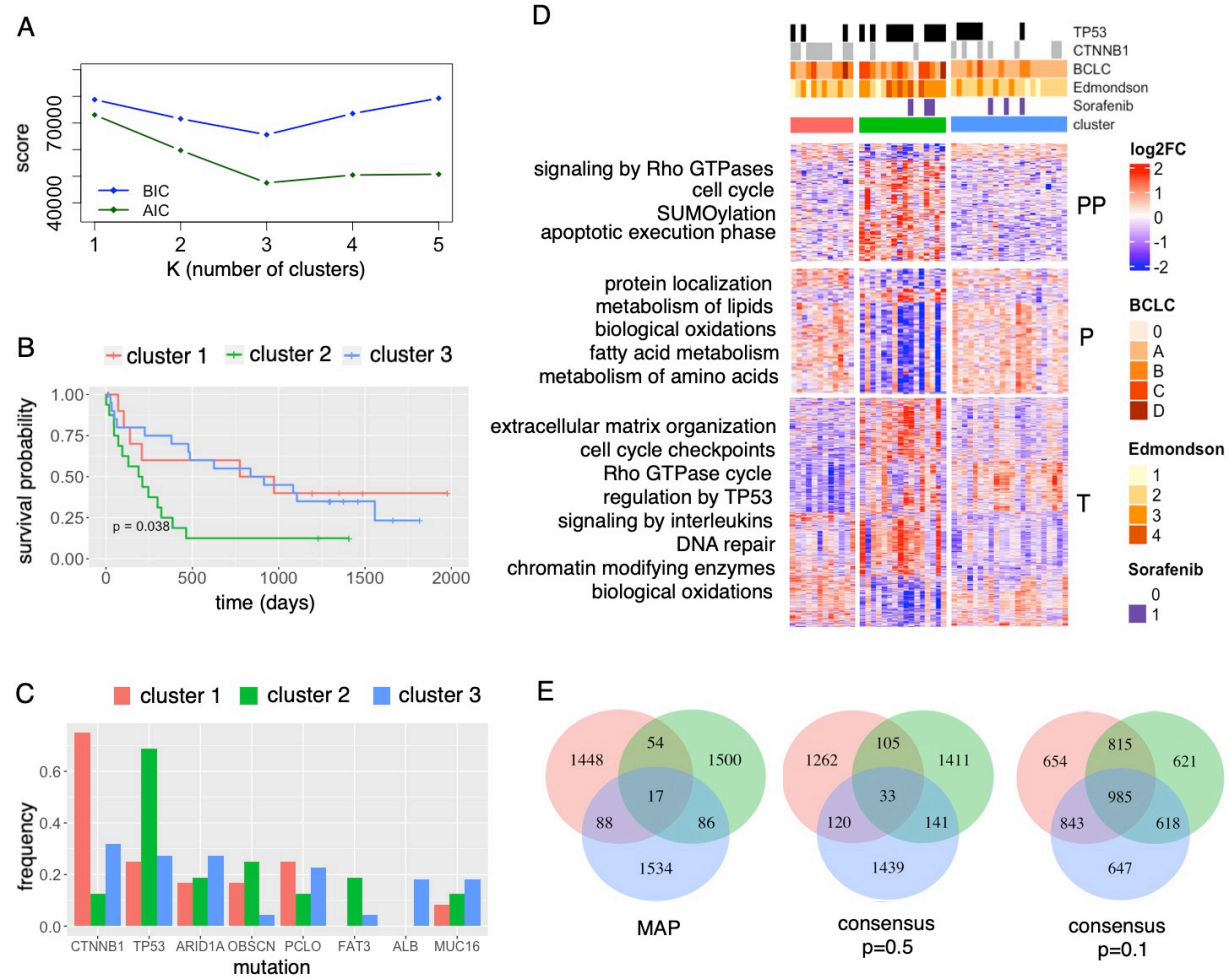
Multi-omics clustering of the HCC dataset with bnClustOmics. (A) BIC and AIC scores of models with different numbers of clusters. (B) Kaplan-Meier survival curves for patients in discovered clusters. (C) Mutational frequencies in discovered clusters. Only mutations with frequency ≥15% in at least one of the clusters are shown. (D) Pathway enrichment differences between clusters. (E) Venn diagrams showing the number of common and cluster-specific edges in the discovered MAP and consensus networks learned for cluster 1 (red), cluster 2 (green), cluster 3 (blue); edge directions were disregarded.

We ran the algorithm for *K* = 1, 2, 3, 4, and 5 clusters. The BIC and AIC scores indicated *K* = 3 as the optimal number of clusters (Fig 4A). *K* = 3 clusters were also found as optimal for the same data in [27] and in another HCC study applying a network-based method to the TCGA HCC dataset [41]. Similar to the clusters discovered in [27], the clusters discovered by bnClustOmics (Fig 4D) are associated with mutations in the genes *TP53* and *CTNNB1*, Edmondson grade, and BCLC stage (*p*-values using Fisher’s exact test are 0.012, 0.001, 0.007, and 0.019, respectively). Cluster 1 is dominated by samples with mutations in *CTNNB1*, and cluster 2 is dominated by samples with mutations in *TP53* (Fig 4C). Cluster 3 is the most heterogeneous in terms of mutations. However, all 4 samples with mutations in *ALB* are in cluster 3.

The three discovered subgroups are associated with patient survival with and without adjustment for BCLC stage (S6 Fig, Section 2.11). In particular, the Cox proportional hazards model revealed that cluster 2 is associated with a poor prognosis (*p* = 0.039 for the non-adjusted model and *p* = 0.024 for the adjusted model), while survival prognoses for cluster 1 and cluster 3 are better and similar. We tested several other approaches for multi-omics clustering, including MOFA, which we used for feature selection. None of the models produced patient subgroups significantly associated with survival when adjusting for BCLC stage (S1 Table, Section 2.11).

To identify processes whose regulation is different between the three patient clusters, we performed DGE and pathway enrichment analysis (Section 2.12). The differences in enriched pathways at all omics levels are more pronounced between cluster 2 and the other clusters (Fig 4D). Significant differences in enriched pathways between cluster 1 and cluster 3 were identified only at the transcriptome level, but not the proteome or phosphoproteome level. However, this situation can result from a combination of noisy data and limitations of pathway enrichment analysis [42].

In order to extend the molecular characterization of the discovered clusters beyond expression levels and mutational frequencies, we analyzed the multi-omics networks that define the clusters. The three MAP networks are very different from each other (Fig 4E). At the same time, the similarities between consensus networks constructed at the edge-wise posterior level of 0.1 are substantially larger (Section 2.5). While 0.1 is a low confidence threshold, the proportion of edges that pass this threshold is around 2% of all non-blacklisted edges for each network. Therefore, the high degree of similarity at the 0.1 level suggests that the posterior landscapes are not as different as the MAP structures. This reflects a high level of modeling uncertainty due to the small effective sample sizes from which the networks were learned. The downside of MAP structures is the inability to account for this uncertainty which can lead to overfitting, as we have seen in simulation studies (Fig 3C).

To address this limitation, we took advantage of the Bayesian approach that we used for structure learning and using several posterior thresholds constructed consensus networks for downstream analysis (Section 2.5).

### 1.4 Downstream effects of mutated genes

bnClustOmics allows for identifying links between genotypes and molecular characteristics of individual clusters. We analyzed all children of *M* (mutation) nodes in the cluster-specific networks. At the first step, we performed pathway enrichment analysis and identified KEGG pathways that are enriched with cluster-specific children of mutation nodes (S2 Table). Signaling pathways associated with HCC, including PIK3-Akt, p53 and cell cycle, were enriched in all clusters. The differences in enriched pathways between the clusters can be connected to their genotypes. For example, the Wnt signaling pathway, whose activation is usually associated with mutations in *CTNNB1* is enriched in cluster 1 and cluster 3 but not in cluster 2. Network *G*_3_ is characterized by more connections than other networks due to a higher level of heterogeneity in cluster 3. As a result, more pathways were found to be enriched with direct neighbors of *M* nodes.

**Fig 5:**
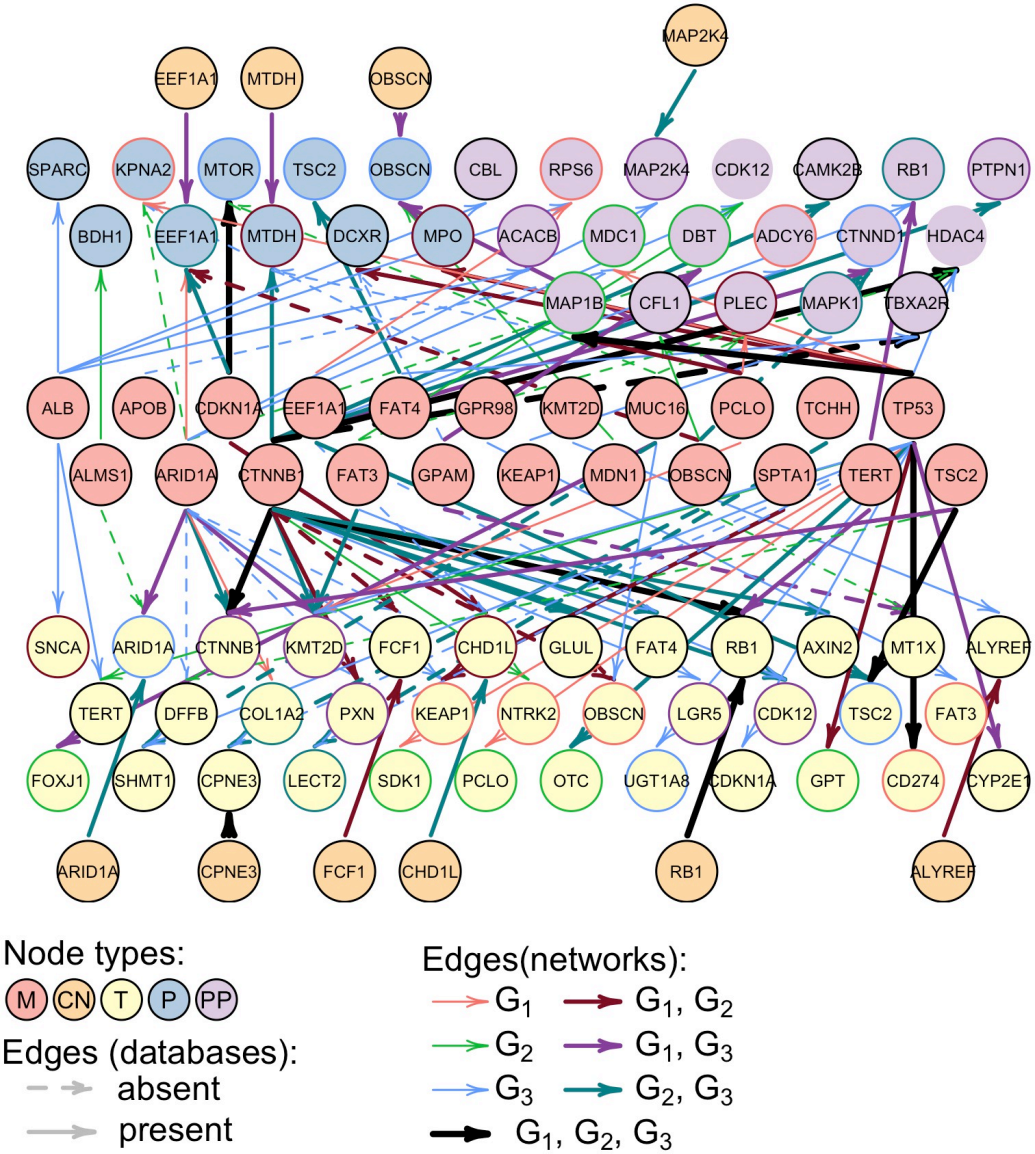
Mutated genes and their most common interaction partners in HCC networks learned by bnClustOmics. Only those *T*, *P*, and *PP* nodes are shown that are differentially expressed/phosphorylated in at least one cluster or the whole dataset. Edges are shown based on their posterior probability: either if they have a high total posterior probability (sum across clusters is at least 1.2), or if they have a high posterior probability in at least one of the clusters (*p* > 0.9). Edge colors indicate in which cluster-specific networks the edges are present with a posterior probability *p* > 0.4: red(*G*_1_), green(*G*_2_), blue (*G*_3_), brown (*G*_1_ and *G*_2_), violet (*G*_1_ and *G*_3_), turquoise (*G*_2_ and *G*_3_), black (*G*_1_ and *G*_2_ and *G*_3_). Border colors of *T*, *P*, and *PP* nodes represent the differential expression status (color scheme is the same as edge colors). Solid edges denote either connections between two omics types of the same gene or interactions found in the STRING and Omnipath databases.

We further scrutinized the individual edges connecting mutations to the nodes representing genes products of other omics types. To find the connections which can explain the abnormal expression of *T*, *P* and *PP* nodes, we have selected children of all mutation nodes in all networks which are differentially expressed in at least one cluster or in the whole dataset (Fig 5). The frequently mutated genes *TP53*, *CTNNB1*, and *ARID1A* have the most children across networks. However, *CTNNB1* has the largest proportion of children that are the same across the clusters, whereas the children of *ARID1A* are rather different across the clusters. This suggests that the effects of mutations in *CTNNB1* are more homogeneous, while effects of *ARID1A* are more heterogeneous across clusters. *ARID1A* is a sub-unit of chromatin remodeling complex SWI/SNF and may have broad effects on gene expression levels. Heterogeneous roles of *ARID1A* in HCC were already pointed out in previous studies [43]. In particular, *ARID1A* was found to act both as a tumor suppressor and oncogene depending on the context.

We noted that bnClustOmics was able to capture some of the well-known HCC-specific interactions while performing *de novo* clustering. One example of homogeneous connections is the edge from mutation in *CTNNB1* (denoted *CTNNB1*-*M*) to the *CTNNB1* transcript abundance (*CTNNB1*-*T*). In all clusters, the mutation status of *CTNNB1* is positively correlated with the expression of the *CTNNB1* transcript. In cluster 1 and cluster 3, *CTNNB1*-*T* is overexpressed compared to normal samples. This corresponds to the known effects of *CTNNB1* mutations in HCC [44]. However, in cluster 2, *CTNNB1*-*T* is not overexpressed, despite the edge between *CTNNB1*-*M* and *CTNNB1*-*T*. This situation results from cluster 2 containing only two samples with mutated *CTNNB1* and the fact that mutations in *TP53* are not associated with increased *CTNNB1*-*T*. This example demonstrates the complementary roles of network analysis with DGE in the downstream analysis.

**Fig 6:**
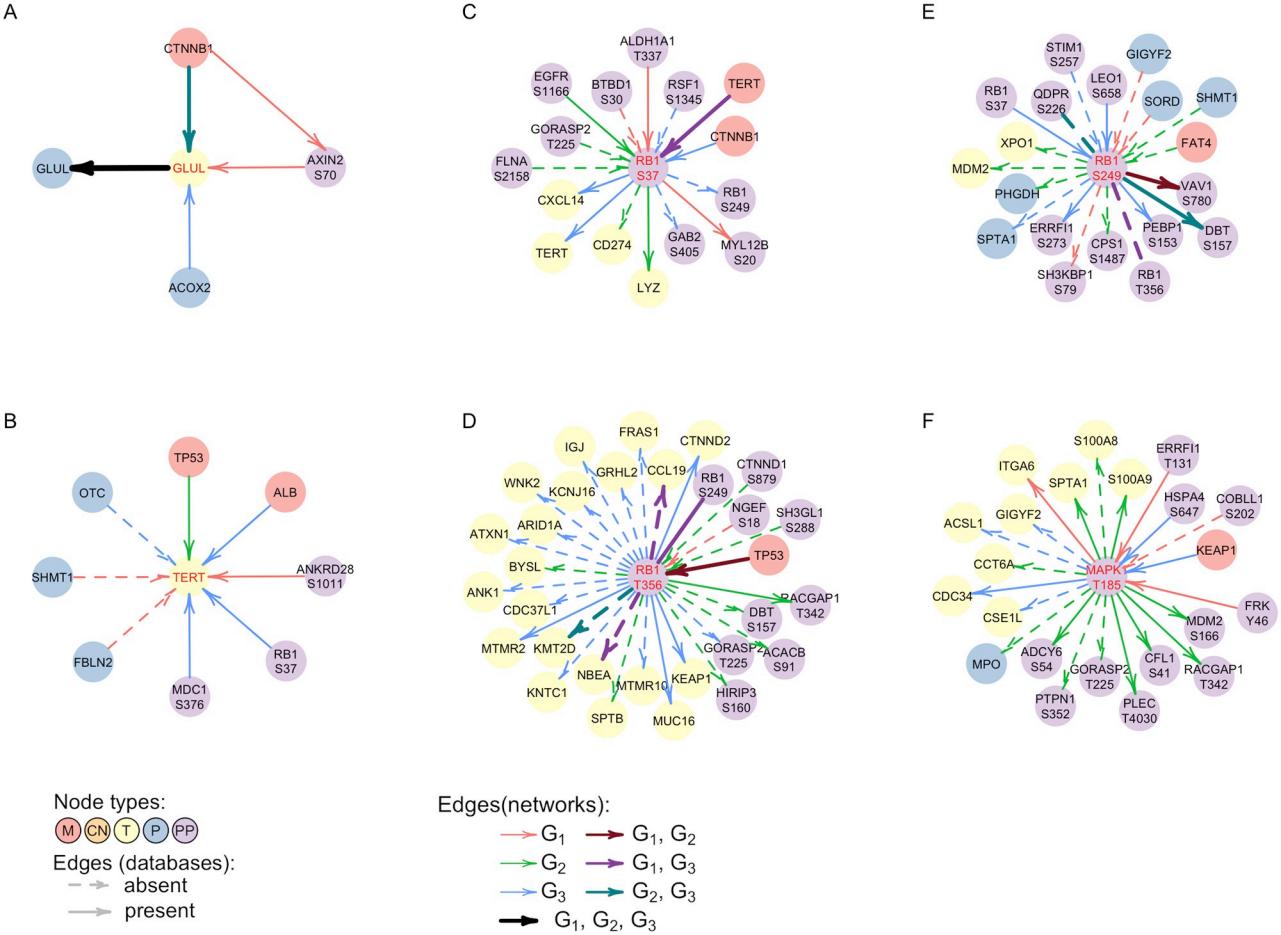
Neighborhoods of individual nodes in the networks learned by bnClustOmics. Direct neighbors of nodes (A) *GLUL*-*T* (B) *TERT*-*T* (C) RB1-S37 (D) RB1_T356 (E) RB1-S249 (F) MAPK1_T185 in multi-omics networks discovered by bnClustOmics. Interactions are only shown between the central node and all of its direct neighbors with exception of (A) where we also show the connection between *CTNNB1*-*M* and AXIN2_S70.

The edge from *CTNNB1*-*M* to *GLUL*-*T* which is present in *G*_2_ and *G*_3_ is another example of a previously known interaction. *GLUL* is known to be upregulated in HCC and is associated with the mutated *CTNNB1*. It is also known that *GLUL* is affected by activation of the Wnt/*β*-catenin pathway at the transcription level, so the incoming edges in the *GLUL*-*T* node are consistent with previous findings [45]. Interestingly, there is no edge connecting *CTNNB1*-*M* and *GLUL*-*T* in *G*_1_. If we examine the interaction partners of *GLUL*-*T* (Fig 6A), there is an incoming edge that is specific to *G*_1_ coming from the phosphorylation site AXIN2_S70, and AXIN2_S70 has an incoming edge from *CTNNB1* also only in *G*_1_. AXIN2, just like *GLUL*, is a known target of the Wnt/*β*-catenin pathway [46]. The link between proteins GLUL and AXIN2 is also present in the STRING database with an interaction score of 0.42. The phosphorylation site AXIN2_S70 has been mentioned in the study connecting mutations to signaling in breast cancer [47]; however, there have been no previous studies about this phosphorylation site in HCC. Thus, the different path from *CTNNB1*-*M* to *GLUL* in *G*_1_ compared to *G*_2_ and *G*_3_ may represent differences in signaling leading to the same target. Alternatively, due to a limited number of observations, we may have captured the same process with a different set of edges, so further experiments are needed to clarify this link.

In addition to edges corresponding to known interaction contexts, bnClustOmics discovered edges pointing at new context-specific dependencies. Cluster 2 is characterized by mutations in the *TP53* gene, and we analyzed *TP53*-*M* connections which might contribute to the phenotype of cluster 2 (S6 Appendix). The transcript node *TERT*-*T* is differentially expressed in cluster 2 and also has an incoming edge from *TP53*-*M* in *G*_2_. *TERT*-*T* expression is known to be upregulated in many cancers including HCC [48] and it is also significantly overexpressed in all clusters in the analyzed cohort. However, the expression level of *TERT*-*T* is significantly higher in cluster 2 than in cluster 1 and cluster 3 (Fig B in S6 Appendix). The high degree of *TERT*-*T* overexpression is associated with mutations in *TP53* as suggested by *G*_2_. At the same time, the edge from *TP53*-*M* to *TERT*-*T* is absent in *G*_1_ and *G*_3_ (Fig A in S6 Appendix), suggesting that the effect of mutated *TP53* on *TERT*-*T* is only present in cluster 2.

We investigated connections of *TERT*-*T* in other networks to identify possible sources of its upregulation in remaining clusters (Fig 6B). There is an incoming edge from ALB-*M* in *G*_3_; however, it is negatively correlated with *TERT*-*T* expression, so mutations in ALB-*M* do not seem to contribute to *TERT*-*T* overexpression. In addition, there is a *G*_3_-specific incoming edge from the phosphorylation site RB1_S37, which is overexpressed in cluster 2 and cluster 3, but not in cluster 1. Network *G*_3_ suggests that RB1_S37 is associated with overexpression of *TERT*-*T* and hence might also contribute to carcinogenesis. In *G*_3_, there is an incoming edge to RB1_S37 from *CTNNB1*-*M* and the corresponding correlation is positive. This suggests that *CTNNB1*-*M* contributes to RB1_S37 overexpression and via RB1_S37 may affect *TERT*-*T* as well. However, this dependency is not direct and weaker than the edge from *TP53*-*M* to *TERT*-*T* in cluster 2, suggesting that the direction of the effect of mutations in *CTNNB1* and *TP53* on the expression of *TERT* is the same, but the effect size is different. This finding aligns with the associations between mutations in *CTNNB1* and *TP53* and survival. Both mutated genes are drivers of HCC however, *TP53* results in a poorer prognosis than *CTNNB1*.

Other RB1 phosphorylation sites, namely S249 and T356, are highly phosphorylated across all clusters. Moreover, we observe several incoming edges from *M* nodes in all RB1 phosphorylation sites (Fig 6C–6E). The mutation statuses of parent nodes of RB1 (*FAT4*-*M* in cluster 2, *TERT*-*M* in cluster 3, *TP53*-*M* in cluster 1) are positively correlated with increased phosphorylation of the respective sites, suggesting that they all may contribute to RB1 hyperphosphorylation. In previous studies, RB1 has been shown to play an important but complex role in cell cycle regulation and apoptosis [49]. It can act both as a tumor suppressor and oncogene depending on its phosphorylation status. All three phosphorylation sites included in our network can be found in the PhosphoSitePlus database [50]. The role of S249 and T356 phosphorylation is well studied and known to affect the cell cycle and apoptosis. The role of S37 phosphorylation is less well known, and there are no studies about its role in HCC. As previously noted, our analysis suggests that phosphorylation of this site may also play a role in HCC. We note that *RB1*-*T* is also overexpressed. However, there are no edges between *RB1*-*T* and RB1 phosphorylation sites (S7(B) Fig), suggesting that overexpression of *RB1*-*T* is not the main source of RB1 hyperphosphorylation. In addition, since unphosphorylated RB1 acts as a tumor suppressor, knocking it down does not seem wise. Many efforts rather target inhibiting its phosphorylation and activating its tumor-suppressive properties [49, 51]. Furthermore, Indovina et al. [49] mention Cdk inhibitors as possible therapies which can prevent RB1 phosphorylation. Indeed, Ng et al. [27] found an association of overactive CDK1/CDK2/CDK5 kinases and the phenotype associated with mutations in *TP53*. The central role of phosphorylation of RB1 in all networks suggests that inhibition of Cdk can be beneficial for patients in all clusters.

Many of the edges in discovered networks are absent in the public PPI databases. The edge from *TP53*-*M* to *LECT2*-*T* is present in *G*_3_, and *TP53*-*M* is negatively correlated with *LECT2*-*T* in this cluster (it is also negatively correlated with *LECT2*-*T* in *G*_2_, but this edge has a low posterior probability). We note that *LECT2*-*T* is also downregulated in cluster 2 and cluster 3, but not in cluster 1. The downregulation of *LECT2*-*T* has been previously associated with a poor prognosis in HCC and mutations in *TP53* [52]. Thus, the discovered link between mutations in *TP53*-*M* and downregulation of *LECT2*-*T* is plausible, despite being absent in the STRING database. We further noted that *LECT2*-*M* has an incoming edge from *TCHH*-*M* in both *G*_2_ and *G*_3_, while *TCHH* mutations are absent in cluster 1. Both *M* nodes are negatively correlated with *LECT2*-*T* suggesting that *TCHH*-*M* contributes in a similar way to the molecular phenotype as *TP53*-*M*. Heterogeneity is a known issue in identifying cancer subtypes. One implication of shared connections of different mutated genes in the discovered networks is that they affect similar downstream genes and may be targeted by similar therapies.

At the same time, some *M* nodes have opposite effects on the same interaction partners, indicating opposite effects of these corresponding mutated genes on the phenotype. *TP53*-*M* and *CTNNB1*-*M* share two common connections: HDAC4_S246 and *KMT2D*-*T*. In both cases, the mutation status of *CTNNB1* and *TP53* are oppositely correlated with their shared interaction partners. The correlation between *TP53*-*M* and *KMT2D*-*T* is positive, while the correlation between *KMT2D*-*T* and other *M* nodes (shown in S7(A) Fig) including *CTNNB1* is negative. In pancreatic cancer, low expression of *KMT2D* has been associated with a better prognosis [53]. Moreover, knock-out of *KMT2D* has been shown to attenuate cell proliferation and was suggested as a therapeutic target [54]. Opposite effects of *TP53*-*M* and *CTNNB1*-*M* on *KMT2D*-*T* in cluster 3 suggest that co-occurrence of these mutations may diverge the phenotype from phenotypes where *TP53* and *CTNNB1* do not co-occur. Mutations in *CTNNB1* and *TP53* have been considered mutually exclusive in many studies [55]. However, they co-occur in 10% of all samples in the analyzed dataset. The mutual exclusivity was also challenged by a study presenting a detailed case of *TP53*/*CTNNB1* co-occurrence in the same tumor [56]. In addition, we observe an interesting pattern of co-occurrence of *TP53* and *CTNNB1* across discovered clusters as four out of five co-occurrence cases fall outside of the *TP53*-dominated cluster 2, which can also hint at possible opposite effects of mutations in *TP53* and *CTNNB1* on the phenotype. Our findings align with another HCC classification based on morphological features of the tumor and gene expression [57]. The analysis by Trobenson et al. Torbenson2021 indicated that *CTNNB1* and *TP53* were associated with opposite effects on the presence of pseudoglands (a histopathologic feature used for HCC characterization in clinics). In addition, the majority of samples with co-occurring *CTNNB1*/*TP53* mutations ended up in the *CTNNB1* cluster based on the gene expression data. However, *CTNNB1*/*TP53* mutated tumors were associated with clonal progression, in contrast to tumors harboring only *CTNNB1*.

### 1.5 Hub phosphorylation sites

In studies devoted to PPI network characterization, the number of neighbors (degree) of a node in the network is often used to characterize its biological importance [58, 59]. The degree distribution of the discovered networks suggests that nodes with more than 20 neighbors can be considered as hubs (S8(A) Fig). Identified hubs score high in terms of betweenness indicating their importance for the biological processes encoded by the networks (S8(B) Fig). In order to investigate the network structure in connection to HCC subtypes, we defined two lists of the most connected nodes. In the first list, we included the top twenty nodes with the largest number of connections that are present with non-zero posterior probabilities in two or all networks (S1 File). Such nodes and their direct neighbors represent the most similar parts between the networks. In the second list, we included all nodes with the largest number of cluster-specific connections (S2 File). Interestingly, the nodes in the first list turned out to be *P* nodes (9 out of 20), *M* nodes (9 out of 20) nodes and *T*-nodes (2 out of 20) while the top nodes of the second list were dominated by *PP* nodes (17 out of 20). Hence, of all omics types, phosphorylation sites appear to have the most different neighborhoods between the clusters. While for *CN*, *T*, and *M* nodes, this can be explained by model structural restrictions, for *P* and *PP* nodes, this finding suggests that differences in the interactome between clusters are more substantial at the phosphoproteome level than at the proteome level. This finding aligns with the analysis of modules of the discovered networks: of the four largest modules, three are dominated by *PP* nodes (S5 Table).

The list of most differentially connected phosphorylation sites includes MAPK1_T185, CTNND1_S252, and GRB14_S372, which are known to play a role in HCC signaling and affect the regulation of cell cycle, apoptosis, and carcinogenesis (S3 Table). Some of these hub-phosphorylation sites have been found to be important in other cancers than HCC, e.g., ANKRD28_S1011, PRKAA2_S491, and TBXA2R_S331. Our networks suggest that they might also play a role in HCC and are thus candidates for further experiments.

MAPK1 is known to be essential for MAP kinase signaling, which is one of the targets of Sorafenib [60–62], a standard-of-care treatment for advanced HCC. The phosphorylation site MAPK1_T185 is increased in cluster 2 and cluster 3 and has a considerable amount of cluster-specific connections in *G*_2_ (Fig 6E). The phosphorylation of another MAPK1 site, namely Y187, is significantly increased in cluster 3 only. Both phosphorylation sites have many references in the PhosphoSitePlus database, and are known to induce carcinogenesis and alter apoptosis, and are known drug targets. However, MAPK1 is known to be active if both sites are phosphorylated [63]. The increased phosphorylation of both sites is observed only in cluster 3. At the same time, the role of mono-phosphorylated MAPK1 is not fully understood [64]. Sorafenib which inhibits upstream regulators of MAPK1 [65] was given to six patients from the analyzed cohort, three of which were assigned to cluster 2 and three to cluster 3. Five out of six patients had to discontinue treatment due to side-effects, but patients from cluster 3 on average tolerated the therapy longer and survived longer than patients who were treated with Sorafenib in cluster 2 (S7 Appendix). This separation aligns well with our clustering, although it is not possible to make stronger conclusions due to a limited number of biopsies and the short duration of treatment.

One of the MAPK1_T185 interaction partners in *G*_2_ is another hub phosphorylation site, PTPN1_S352, whose phosphorylation is increased in cluster 2 only. *PTPN1* is known to play an important role in many liver diseases; however, it can act both as a tumor suppressor, and oncogene in HCC [66]. Most studies suggest its tumor-suppressive role. However, our analysis indicates that increased phosphorylation of PTPN1_S352 is associated with a poor prognosis and increased phosphorylation of MAPK1_T185 in cluster 2. This connection is confirmed in [67], where *PTPN1* was identified as an oncogene, and its knockdown resulted in attenuated Ras activity and MAPK signaling. We found several inhibitors of PTPN1 in The International Union of Basic and Clinical Pharmacology (IUPHAR) / British Pharmacological Society (BPS) Guide to PHARMACOLOGY [68]. All of them have hypoglycaemic and other anti-diabetic effects. Previous studies already pointed out the anti-tumor properties of diabetes drugs on HCC [69]. We believe that investigating strong individual dependencies in cluster-specific networks coupled with DGE might suggest drug candidates and highlight interactions that are important in the context of different subtypes of HCC.

### 1.6 Discussion

Learning biological networks and cancer subtyping based on multi-omics molecular data are challenging problems, which are traditionally addressed by separate computational methods. In this work, we present bnClustOmics, a tool that tackles both problems simultaneously. Our approach can integrate and cluster multi-omics datasets and learn networks consisting of different types of omics variables, each of which characterizes a patient cluster. In simulation studies, we have shown that bnClustOmics outperforms other clustering approaches due to its ability to detect differences in network structures, while other algorithms mostly lack this ability. A major limitation of our method is the necessity to perform feature selection, which is not straightforward in an unsupervised setting. We suggest using a combination of MOFA and DGE analysis based on our simulation studies, but other ways can also be explored in the future. The package bnClustOmics can be applied to any combination of omics types and is not limited to the five omics types analyzed in this HCC cohort. In the current implementation, there is no possibility to learn the edges between discrete nodes. This feature can further refine clustering, but it makes sense only for larger datasets due to the extreme sparsity of the mutation data.

We applied bnClustOmics to an HCC dataset comprising five different omics types. Similar to previous studies [27, 41, 57], the three discovered clusters are associated with mutations in *CTNNB1* and *TP53*, and the BCLC stage. Our patient clustering is significantly associated with survival with and without adjustment for the BCLC stage. Cluster 2 is dominated by samples with mutated *TP53* and is associated with a poor prognosis. Samples in which *CTNNB1* and *TP53* co-occur are mostly found in cluster 1 and cluster 3. Moreover, we find that *CTNNB1* and *TP53* have opposite effects on the expression of the transcript *KMT2D* and the phosphorylation site HDAC4_S246 in the learned networks. These findings might explain why *CTNNB1* and *TP53* show mutual exclusivity patterns [70, 71] and are associated with opposite effects of the phenotype [57] in some cohorts.

On a more general level, our analysis suggests that the discovered clusters are associated with changes in signaling networks as identified by substantial differences in the neighborhoods of phosphorylation sites. The differences between interactions partners are the largest on the phosphoproteome level, suggesting that this omics type brings a major contribution to the result of the network-based clustering highlighting the importance of phosphoproteome data for further studies.

Cluster-specific networks suggest that hyperphosphorylation of RB1 is associated with mutations in *TP53*, *CTNNB1*, and *FAT4* but not with overexpression of *RB1* at the transcriptome level. This finding aligns with previous studies suggesting that unphosphorylated RB1 acts as a tumor suppressor, while hyperphosphorylation of RB1 contributes to carcinogenesis [49]. Hence therapies that inhibit phosphorylation of RB1 such as Cdk inhibitors may be a promising treatment strategy.

Overall, our analysis has shown that including associations between different omics types in the clustering model is an important step towards defining cancer subtypes and their molecular makeup comprehensively. These novel associations may improve the selection of effective personalized therapies.

## 2 Methods

### 2.1 Data

We applied bnClustOmics to the HCC data analyzed in [27]. The full dataset comprises 51 biopsies from 49 patients with HCC diagnosis. For each patient, DNA, RNA, proteome, and phosphoproteome data are available. For two patients, two sets of biopsies were available from two genetically different HCC tumors. In addition, we obtained data from 15 biopsies from healthy livers for transcriptome analysis and 11 biopsies for proteome and 10 for phosphoproteome analysis from the same study. A detailed description of sequencing, library preparation, transcript quantification, and SWATH analysis can be found in [27]. We obtained the normalized data from Ng et al. [27] and performed data imputation and batch-correction where applicable (S5 Appendix). One sample was hypermutated with over 9000 mutated genes and was excluded from the analysis. Consequently, we included 50 biopsies from 48 patients in the study.

### 2.2 Bayesian network mixture model

We assume that the data *D* consisting of *N* observations is generated from a mixture of *K* components with weights *τ*_*k*_. Each component is a Bayesian network $B_{k}$, a directed probabilistic graphical model representing a factorization of the joint distribution of the random variables *X*_1_, …, *X*_*n*_. The random variables are used to model omics features in the analyzed dataset (*M*, *CN*, *T*, *P* and *PP*). Each patient sample *D*_*i*_ represents a vector of *n* values (one for each *X*_*j*_) and is generated from a model $B_{k}$, depending on the value of a hidden variable *Z*_*i*_ [14],

$$\begin{array}{c} D_{i}|\left(Z_{i}=k\right)\;\;∼\;\;B_{k}=\left(G_{k},\theta_{k}\right), \end{array}$$ (1)

where *G*_*k*_ is a DAG and *θ*_*k*_ are the parameters of the local probability distributions (LPD).

A Bayesian network mixture model was first suggested in [14] for (single-omics) binary mutation data. In our model, each network consists of binary (mutations), ordinal (CNA), and continuous variables (transcriptome, proteome, and phosphoproteome). We denote the set of indices of all binary, ordinal, and continuous nodes by Ω, Φ, and Ψ, respectively. The quantities *n*_*b*_, *n*_*o*_, and *n*_*c*_ are the numbers of binary, ordinal, and continuous random variables, respectively, in the network. We model the LPD for each continuous node *X*_*ψk*_, *ψ* ∈ Ψ, of each mixture component by linear regression on its parents in graph *G*_*k*_,

$$\begin{array}{c} P\left(X_{\psi k}|{\text{Pa}}_{\psi k},\;\theta_{k},\;G_{k}\right)\;\;=\;\;N(X_{\psi k}\;\;|\;\;\;m_{\psi k}+\sum_{X_{\xi \;k}\in {\text{Pa}}_{\psi k}}\beta_{\psi k}^{\xi }X_{\xi \;k},\;\;\sigma_{\psi k}^{2}), \end{array}$$ (2)

where **Pa**_*ψk*_ is the set of parents of node *X*_*ψk*_ in graph *G*_*k*_. The set of parameters of the LPDs of continuous nodes includes a vector of regression intercepts *m*_*k*_, a vector of standard deviations *σ*_*k*_, and a vector of regression coefficients *B*_*ψk*_ defined for all nodes with non-empty parent set. Given a graph *G*_*k*_, the Gaussian Bayesian network model above can be equivalently parameterized using a vector of unconditional means *μ*_*k*_ and a covariance matrix Σ_*k*_ (Section 1 in S4 Appendix). We use both parametrizations interchangeably. Binary and ordinal nodes are not allowed to have parents by assumption. For binary nodes *X*_*ωk*_, we assume that the LPDs are defined by the parameters

$$\begin{array}{c} \lambda_{\omega k}=P\left(X_{\omega k}=1\right) \end{array}$$ (3)

and for ordinal nodes *X*_*ϕk*_, we use the Gaussian approximation

$$\begin{array}{c} P\left(X_{\phi k}|\theta_{k}\right)\;\;=\;\;N\left(X_{\phi k}|m_{\phi k},\;\sigma_{\phi k}^{2}\right). \end{array}$$ (4)

We denote the set of all parameters of a mixture component *k* by *θ*_*k*_ = (*λ*_*k*_, *μ*_*k*_, Σ_*k*_).

### 2.3 EM algorithm

Following [14] we use an EM algorithm for learning Bayesian network mixture models. We denote by *D*_*i*_ the *i*-th observation in the dataset, representing a vector of omics measurements of one patient (or one biopsy in case of multiple biopsies per one patient). The algorithm proceeds as follows:

1. Initialize cluster membership probabilities *γ*_*ik*_ of patient *i* being in cluster *k* (Section 2.7)
2. Given *γ*_*ik*_, perform MAP structure search and estimate DAGs ${\hat{G}}_{k}$ (Section 2.5)
3. Given estimated DAGs ${\hat{G}}_{k}$, iterate *q* times:
  - (M-step) Compute MAP parameters ${\hat{\theta }}_{k}$ (Section 2 in S4 Appendix)
  - (E-step) Update membership weights
    $$\gamma_{ik}=\frac{\tau_{k}P\left(D_{i}|{\hat{G}}_{k},{\hat{\theta }}_{k}\right)}{\sum_{k^{\}}=1}^{K}\tau_{k^{\}}}P\left(D_{i}|{\hat{G}}_{k^{\}}},{\hat{\theta }}_{k^{\}}}\right)}$$
    and cluster weights
    $$\tau_{k}=\frac{\sum_{i=1}^{N}\gamma_{ik}}{N}$$
    (Section 2.6)
4. Iterate steps 2 and 3 until convergence

The internal cycle with *q* iterations is added for computational efficiency because parameter updates are computationally less expensive than structure search. Hence, for each update of the structures, we perform *q* updates of the parameters. We learn cluster membership assignments for all patients *D*_*i*_ and MAP networks ${\hat{G}}_{k}$. Once the EM algorithm has converged, bnClustOmics can optionally perform sampling from the posterior distribution and the output includes the matrices of estimated probabilities of all edges (Section 2.5).

The main differences to the procedure in [14] are a different set of parameters *θ*_*k*_ and network structural constraints due to the multi-omics extension and differences in data types.

### 2.4 Network score

For assessing how well the network structure fits the data, we use the BGe score [72, 73]. In addition to the model assumption specified in Eq 2, the BGe score requires technical assumptions on likelihood and parameter prior [72]. The network score *R*(*G*_*k*_∣*D*) then decomposes over continuous nodes as

$$\begin{array}{c} P\left(G_{k}|D\right)\propto R\left(G_{k}|D\right)=\prod_{\psi \in \Psi }S\left(X_{\psi k},{\text{Pa}}_{\psi k}|D\right). \end{array}$$ (5)

By our model design, nodes *X*_*ϕ*_ and *X*_*ω*_, corresponding to mutations and copy number changes, are not allowed to have any parents. Hence, the terms *S*(*X*_*ϕk*_, **Pa**_*ϕk*_∣*D*) = *S*(*X*_*ϕk*_∣*D*) and *S*(*X*_*ωk*_, **Pa**_*ϕk*_∣*D*) = *S*(*X*_*ωk*_∣*D*) are constant for all possible graphs. For this reason, we exclude these terms when performing structure search and the product in Eq 5 runs only over nodes *X*_*ψk*_. However, nodes *X*_*ϕk*_ and *X*_*ωk*_ may enter the equation as parents of *X*_*ψk*_.

### 2.5 Structure search

At each step of structure search, we use the iterative order MCMC scheme introduced in [31] and implemented in the R-package BiDAG [32], which proved to be superior to many other methods for MAP structure search in simulation studies [31]. An optional step after the MAP graph has been found is to sample graphs from the posterior distribution using the order MCMC scheme [31]. This step allows us to estimate consensus models by averaging over a sample of *L* graphs from the posterior distribution. In particular, the posterior probability of an edge *e*_*ξψk*_ between nodes *X*_*ξk*_ and *X*_*ψk*_ in the graph *G*_*k*_ is estimated as:

$$\begin{array}{c} P\left(e_{\xi \psi k}|D\right)\approx \frac{1}{L}\sum_{l=1}^{L}1\left\{e_{\xi \psi k}\in G_{k}^{l}\right\}, \end{array}$$ (6)

where $1\{e_{\xi \psi k}\in G_{k}^{l}\}$ = 1 if the edge *e*_*ξψk*_ is present in structure $G_{k}^{l}$ and 0 otherwise. Edges whose posterior probabilities are lower than a defined posterior threshold are excluded from the resulting consensus structure [31].

We use the iterative MAP search at the second step of the EM algorithm and perform sampling once after the EM has converged to compute posterior probabilities of single edges and identify consensus graphs.

To construct graphs for the downstream analysis, we made a list of edges whose posterior *P*(*e*_*ξψk*_∣*D*) is higher than 0.9 for at least one cluster *k* (the threshold was chosen based on our simulation studies). In addition, we selected all edges whose sum of posteriors in all clusters $\sum_{k=1}^{K}P\left(e_{\xi \psi k}|D\right)>1.2$, while the threshold for individual networks is lower: *P*(*e*_*ξψk*_∣*D*) > 0.5 for at least one cluster *k*. Finally, we constructed the graphs *G*_*k*_ by including edges from the selected list if their posterior *P*(*e*_*ξψk*_∣*D*) > 0.4. The reason behind this selection process is finding high-confidence cluster-specific interactions while not dismissing similarities at lower (but non-zero) posterior levels.

### 2.6 Cluster membership weights

Updating the membership weights *γ*_*ik*_ requires assessment of the likelihoods $P(D_{i}|{\hat{G}}_{k},{\hat{\theta }}_{k})$. The decomposition provided by the Bayesian network model allows us to integrate discrete and continuous data types in measuring how well an observation *D*_*i*_ (a vector consisting of *n*_*c*_ continuous, *n*_*o*_ ordinal, and *n*_*b*_ binary components) fits a DAG ${\hat{G}}_{k}$ and parameters ${\hat{\theta }}_{k}=\left({\hat{\lambda }}_{k},\;{\hat{\mu }}_{k},\;{\hat{\Sigma }}_{k}\right)$:

$$\begin{array}{c} P\left(D_{i}|{\hat{G}}_{k},{\hat{\theta }}_{k}\right)=\prod_{\psi \in \Psi }P\left(D_{i\psi }|{\text{Pa}}_{\psi k},{\hat{\mu }}_{k},{\hat{\Sigma }}_{k}\right)\prod_{\phi \in \Phi }P\left(D_{i\phi }|{\hat{\mu }}_{k},{\hat{\Sigma }}_{k}\right)\prod_{\omega \in \Omega }P\left(D_{i\omega }|{\hat{\lambda }}_{\omega k}\right) \end{array}$$ (7)

The detailed formulas for computing the likelihoods are given in Section 3 in S4 Appendix. We have extended the R-package BiDAG, such that the function scoreagainstDAG is able to accommodate mixed data.

### 2.7 Starting membership weights

In general, the EM algorithm does not guarantee finding the global maximum, and the local maximum it finds will depend on the starting point. For this reason, we use a non-random starting point in order to start in a parameter region of high likelihood and help mitigate the local optima issue. By default (and for the HCC data), the starting cluster membership of patients is defined via running mclust on the first *K* + 2 principal components after applying PCA to the original data. Our simulation studies have shown that dimension reduction via PCA as a starting point improves the results of mclust. The initial membership weights are then defined as

$$\begin{array}{c} \gamma_{ik}=\{\begin{array}{cc} \frac{3}{K+2}, & \text{if}\;k=g_{i}\\ \frac{1}{K+2}, & \text{otherwise,} \end{array} \end{array}$$ (8)

where *g*_*i*_ denotes the cluster assignment of the *i*^*th*^ observation by mclust. PCA is applied only to define the initial membership weights, but the EM algorithm is then applied to original non-reduced data. With a non-random starting point, by default, bnClustOmics runs the EM only once (the results of simulation studies are shown for one run). However, for the HCC dataset, we restarted the EM three times and selected the model with the highest likelihood for each value of *K*

### 2.8 Allowed edges

By design, bnClustOmics only prohibits incoming edges to discrete nodes. In the HCC data analysis, we added more constraints to obtain more biologically relevant networks. The general flow of the information is directed from the DNA to RNA and (pshospho)protein nodes (S4 Table).

Naturally, we allow all possible edges between *P* and *PP* nodes. We do not allow edges between transcripts because the transcripts do not interact directly. When proteome data is not available, it makes sense to approximate protein-protein interactions with transcript-transcript interactions. However, since we have (phospho)proteome data available, we prefer to explain dependencies with more relevant and interpretable edges between (phospho)proteins and between transcripts and proteins.

### 2.9 Edge penalization matrix

When performing structure search, we use the prior information about interactions between the genes included in the networks, following the methodology described by Kuipers et al. [14]. To do this, we modify the default prior distribution over structures *P*(*G*_*k*_) and replace it with

$$P^{\prime }\left(G_{k}\right)\propto \frac{1}{\prod_{\psi \in \Psi }\prod_{\xi :X_{\xi k}\in {\text{Pa}}_{\psi k}}\kappa_{\xi \psi }},$$

where *κ*_*ξψ*_ defines the penalization factor of the edge *X*_*ξk*_ → *X*_*ψk*_. Note that *κ*_*ξψ*_ ≥ 1 and these factors do not depend on *k* since prior knowledge does not include cluster assignments. The change of prior leads to replacing of the score terms *S*(*X*_*ψk*_, **Pa**_*ψk*_∣*D*) with the terms *S*′(*X*_*ψk*_, **Pa**_*ψk*_∣*D*) in Eq 2.4 for all nodes *X*_*ψk*_ with non-empty parent sets:

$$S^{\prime }\left(X_{\psi k},{\text{Pa}}_{\psi k}|D\right)=\frac{S(X_{\psi k},{\text{Pa}}_{\psi k}|D)}{\prod_{\xi :X_{\xi k}\in {\text{Pa}}_{\psi k}}\kappa_{\xi \psi }}.$$

We use the STRING v.11.0 [36] and Omnipath [40] databases to define penalization factors. We penalize the edges by a factor of 2 if they are not found in the databases. The edges corresponding to interactions from the Omnipath database are not penalized. The edges corresponding to the interactions from the STRING database are not penalized if the interaction score is equal to or bigger than 0.5. Otherwise the penalization factor is defined as 2 − 2 * *interaction*_*score*. In addition, we do not penalize the edges between the same genes of different omics types, e.g., the edges *TP53*-*T* → *TP53*-*P* and *TP53*-*CN* → *TP53*-*T* are not penalized.

### 2.10 Feature selection

The structure search is the most computationally expensive step of the learning procedure. The complexity of the structure search scheme depends only on the number *n*_*c*_ of continuous nodes in the network (since the product in Eq 5 goes only over continuous nodes) and equals $O(n_{c}^{3}\;\text{log}\;n_{c})$ [31]. Hence, for the feasibility of bnClustOmics, we must pre-select the features which we include in the Bayesian networks. Another beneficial point of sensible feature selection is better interpretability since the qualitative analysis is hardly possible for networks with thousands of nodes.

We selected 778 omics features in total (Table A in S3 Appendix): 24 *M*, 292 *CN*, 188 *T*, 116 P and 158 *PP*. The main idea behind our feature selection approach was to combine methods that proved to work best in simulation studies (S2 Appendix) with prior knowledge about genes and interactions that are known to be important in HCC signaling (S3 Appendix). In addition to listed criteria we used reasonable filters for selected features: we included only those *M* nodes which are present in at least two samples and *CN* nodes with non-zero variance.

### 2.11 Survival analysis

To study the association of clusters with clinical outcomes, we used the Cox proportional hazards model with and without adjustment for clinical stage BCLC. Time was measured in days from the date of diagnosis. In the adjusted model, we excluded BCLC group “0” consisting of one sample, which did not include death events. If two or more biopsies were available for one patient, one of them was included in the analysis if the cluster assignments for all of them were the same. Otherwise, all samples from the patient were excluded. Two samples of patients who were lost-to-followup were considered censored. We used a likelihood ratio test based on the *χ*^2^ distribution to assess the model fit.

### 2.12 Enrichment analysis

Pathway enrichment analysis was performed using the R package ReactomePA [74]. For each omics type, a list of differentially expressed/phosphorylated genes (proteins, phosphoproteins) with FDR adjusted *p*-value smaller than 0.05 was used as input. Pathways enriched with FDR-adjusted *p*-value smaller than 0.05 were selected for visualization.

### 2.13 Differential gene and protein expression analysis

For DGE analysis, we used the R package edgeR [75] for transcriptome data, and limma [76] for proteome and phosphoproteome data. Genes were considered differentially expressed if the FDR-adjusted *p*-value was smaller than 0.05. For variable selection, we compared tumor to healthy samples for all omics types. For the heatmap in Fig 4D, we compared samples in a specific cluster to samples in all other clusters. In the downstream analysis, we have also performed DGE analysis between tumor samples in individual clusters and healthy samples.

## Supporting information

S1 FigClustering accuracy simulation: Precision.50 Bayesian network mixtures were generated for each simulation setting. For general clustering approaches, the dimension was reduced by applying PCA and running clustering on the first 5 principal components. All integrative multi-omics approaches were applied to the original data unless specified otherwise. CIMLRco denotes clustering results of the application of CIMLR to a subset of data consisting of observations of only continuous variables. $N_{Z_{k}}$ denotes the number of observations in one cluster, *K* the number of clusters, *n*_*c*_ number of continuous nodes, *n*_*b*_ number of binary nodes in networks. (A) *K* = 3, *n*_*c*_ = 100, *n*_*b*_ = 20, $N_{Z_{k}}=200$ (B) *K* = 3, *n*_*c*_ = 100, *n*_*b*_ = 20, $N_{Z_{k}}=20$ (C) *n*_*c*_ = 100, *n*_*b*_ = 20, $N_{Z_{k}}=20$, *K* ∈ {3, 5, 7, 9}; distance between centers set to medium (D) *K* = 3, *n*_*c*_ = 1000, *n*_*b*_ = 100, $N_{Z_{k}}=20$, algorithms were applied to the full data and a subset of data consisting of all binary nodes with non-zero standard deviation and 150 selected continuous nodes; distance between centers set to medium.(PNG)

S2 FigClustering accuracy simulation: F1.50 Bayesian network mixtures were generated for each simulation setting. For general clustering approaches, the dimension was reduced by applying PCA and running clustering on the first 5 principal components. All integrative multi-omics approaches were applied to the original data unless specified otherwise. CIMLRco denotes clustering results of the application of CIMLR to a subset of data consisting of observations of only continuous variables. $N_{Z_{k}}$ denotes the number of observations in one cluster, *K* the number of clusters, *n*_*c*_ number of continuous nodes, *n*_*b*_ number of binary nodes in networks. (A) *K* = 3, *n*_*c*_ = 100, *n*_*b*_ = 20, $N_{Z_{k}}=200$ (B) *K* = 3, *n*_*c*_ = 100, *n*_*b*_ = 20, $N_{Z_{k}}=20$ (C) *n*_*c*_ = 100, *n*_*b*_ = 20, $N_{Z_{k}}=20$, *K* ∈ {3, 5, 7, 9}; distance between centers set to medium (D) *K* = 3, *n*_*c*_ = 1000, *n*_*b*_ = 100, $N_{Z_{k}}=20$, algorithms were applied to the full data and a subset of data consisting of all binary nodes with non-zero standard deviation and 150 selected continuous nodes; distance between centers set to medium.(PNG)

S3 FigClustering accuracy simulation: Strength of signal.50 Bayesian network mixtures were generated for each simulation setting. *K* = 3, *n*_*c*_ = 1000, *n*_*b*_ = 100, $N_{Z_{k}}=20$, algorithms were applied to the full data and a subset of data consisting of all binary nodes with non-zero standard deviation and 150 selected continuous nodes; distance between centers set to medium. Distances between cluster centers are regulated by two parameters: SHD between networks in different clusters divided by the number of edges in one network (*η*) and the proportion of nodes with non-equal means between clusters (*δ*). (A) *η* = 0.1, *δ* = 0.00 (B) *η* = 0.2, *δ* = 0.03 (C) *η* = 0.2, *δ* = 0.04 (D) *η* = 0.3, *δ* = 0.05.(PNG)

S4 FigDefining the optimal number of clusters with bnClustOmics.30 Bayesian network mixtures were generated for each number of clusters *K* ∈ {3, 5, 7, 9} (ground truth). bnClustOmics was applied for each estimated *K* ∈ {1, …, 11} to each generated dataset and $\hat{K}$ was determined by minimizing the AIC or BIC score. The simulation was performed with two values for the number of observations (A) $N_{Z_{k}}=20$ (B) $N_{Z_{k}}=200$.(PNG)

S5 FigSHD between estimated graphs and the ground truth.50 BN mixtures were generated with unequal mixture weights: $N_{Z_{1}}=150$, $N_{Z_{2}}=100$, $N_{Z_{3}}=50$, $N_{Z_{4}}=20$ (cluster 1, cluster 2, cluster 3 and cluster 4). Distance between cluster centers is set to medium. bnClustOmics was used for clustering. The output MAP and consensus structures were compared to the ground truth CPDAG.(PNG)

S6 Fig Hazard ratios. Hazard ratios of discovered clusters without (A) and with (B) adjustment for the BCLC stage.
(PNG)

S7 Fig Connections of *KMT2D* and *RB1* transcripts in networks discovered by bnClustOmics. (A) connections of the node *KMT2D*-T (B) connections of the node *RB1*-T.
(PNG)

S8 Fig Network characterization. (A) Degree distribution of the network consisting of edges from all clusters for which one of the two requirements holds: the sum of posteriors of this edge in all clusters is grater than 1.2 or its posterior in one of the clusters is greater than 0.9. Red bars indicate nodes whose degree is over 20. (B) The betweenness of hub nodes and all other nodes.
(PNG)

S1 Appendix Generating Bayesian network mixtures and data in simulations studies. (PDF)

S2 Appendix Feature selection simulation study. (PDF)

S3 Appendix Feature selection for the HCC analysis. (PDF)

S4 Appendix Further details on linear Gaussian and mixed Bayesian networks. (PDF)

S5 Appendix Data pre-processing. (PDF)

S6 Appendix High-confidence connections of *TP53*-*M* in *G*_2_. Fig A. TP53- node and its neighbors in networks representing three clusters identified by bnClustOmics. Fig B. Log2-fold changes between expression of TERT- in three HCC clusters and mean expression of TERT- in 15 healthy livers.
(PDF)

S7 Appendix Responses to treatment with Sorafenib. (PDF)

S1 Table Cox model fit. Summary of the likelihood ratio test for Cox proportional hazards models based on assignments obtained by clustering algorithms. The models were fitted for *k* = 3 as found optimal by bnClustOmics or for other *k* that was found optimal by method-specific tools or the elbow method when no such tool was available. For all algorithms apart from bnClustOmics and MOFA, all available omics features were used as input. For MOFA, standard deviations filters (1 for *P* features, 2 for *T* and *PP*, 0.5 for *CN* features) were applied as recommended by the authors of the method. Models with the number of clusters found by each model-specific method are marked with * in the column ‘best’.
(PDF)

S2 Table Signaling pathways enriched with direct interactors of *M* nodes in networks discovered by bnClustOmics. FDR values reflect the enrichment of KEGG signaling pathways with children of *M* nodes in cluster-specific networks. FDR values below 0.05 suggest significant enrichment.
(PDF)

S3 Table Known functions of most connected phosphorylation sites. A list of phosphorylation sites with more than 15 cluster-specific interaction partners and their known functions in HCC and other cancers according to the PhosphoSitePlus database.
(PDF)

S4 Table Allowed edges between features in the HCC analysis. Allowed edges (i.e., not blacklisted) between and within omics types in the HCC analysis. Let X and Y denote gene names. Then, all edges from *CN* nodes to *P* nodes of the same genes are encoded as from X-*CN* to X-*P*. Edges between any two genes are encoded as edges between X-*CN* and Y-*P* (this includes the case when X equals Y).
(PDF)

S5 Table Largest modules in the joint network discovered by bnClustOmics. The network consists of edges from all clusters for which one of the two requirements holds: the sum of posteriors of this edge in all clusters is grater than 1.2 or its posterior in one of the clusters is greater than 0.9. The modules were identified by the function cluster_edge_betweenness from the package igraph [77].
(PDF)

S1 File Top twenty most similarly connected nodes and their interactions partners in cluster-specific networks. (CSV)

S2 File Top twenty most differently connected nodes and their interactions partners in cluster-specific networks. (CSV)
