# Supplementary figures and images for: Multi-omics subtyping of hepatocellular carcinoma patients using a Bayesian network mixture model
Source: PLoS Comput Biol. 2022 Sep 6;18(9):e1009767. doi: 10.1371/journal.pcbi.1009767 (PMC9481159; doi:10.1371/journal.pcbi.1009767)

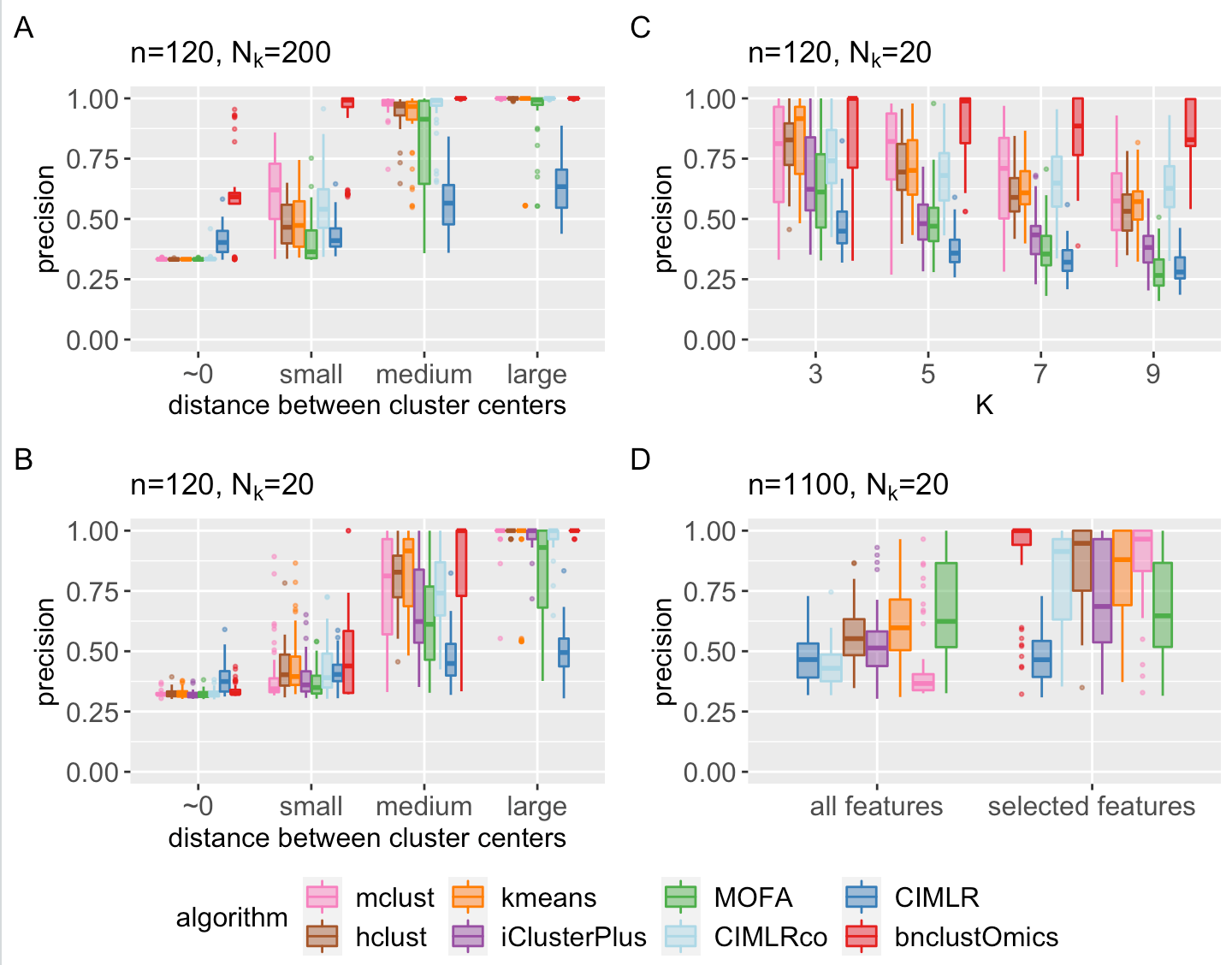

Supplement: S1 Fig — 50 Bayesian network mixtures were generated for each simulation setting. For general clustering approaches, the dimension was reduced by applying PCA and running clustering on the first 5 principal components. All integrative multi-omics approaches were applied to the original data unless specified otherwise. CIMLRco denotes clustering results of the application of CIMLR to a subset of data consisting of observations of only continuous variables. NZk denotes the number of observations in one cluster, K the number of clusters, nc number of continuous nodes, nb number of binary nodes in networks. (A) K = 3, nc = 100, nb = 20, NZk=200 (B) K = 3, nc = 100, nb = 20, NZk=20 (C) nc = 100, nb = 20, NZk=20, K ∈ {3, 5, 7, 9}; distance between centers set to medium (D) K = 3, nc = 1000, nb = 100, NZk=20, algorithms were applied to the full data and a subset of data consisting of all binary nodes with non-zero standard deviation and 150 selected continuous nodes; distance between centers set to medium. (PNG) [file pcbi.1009767.s001.png]

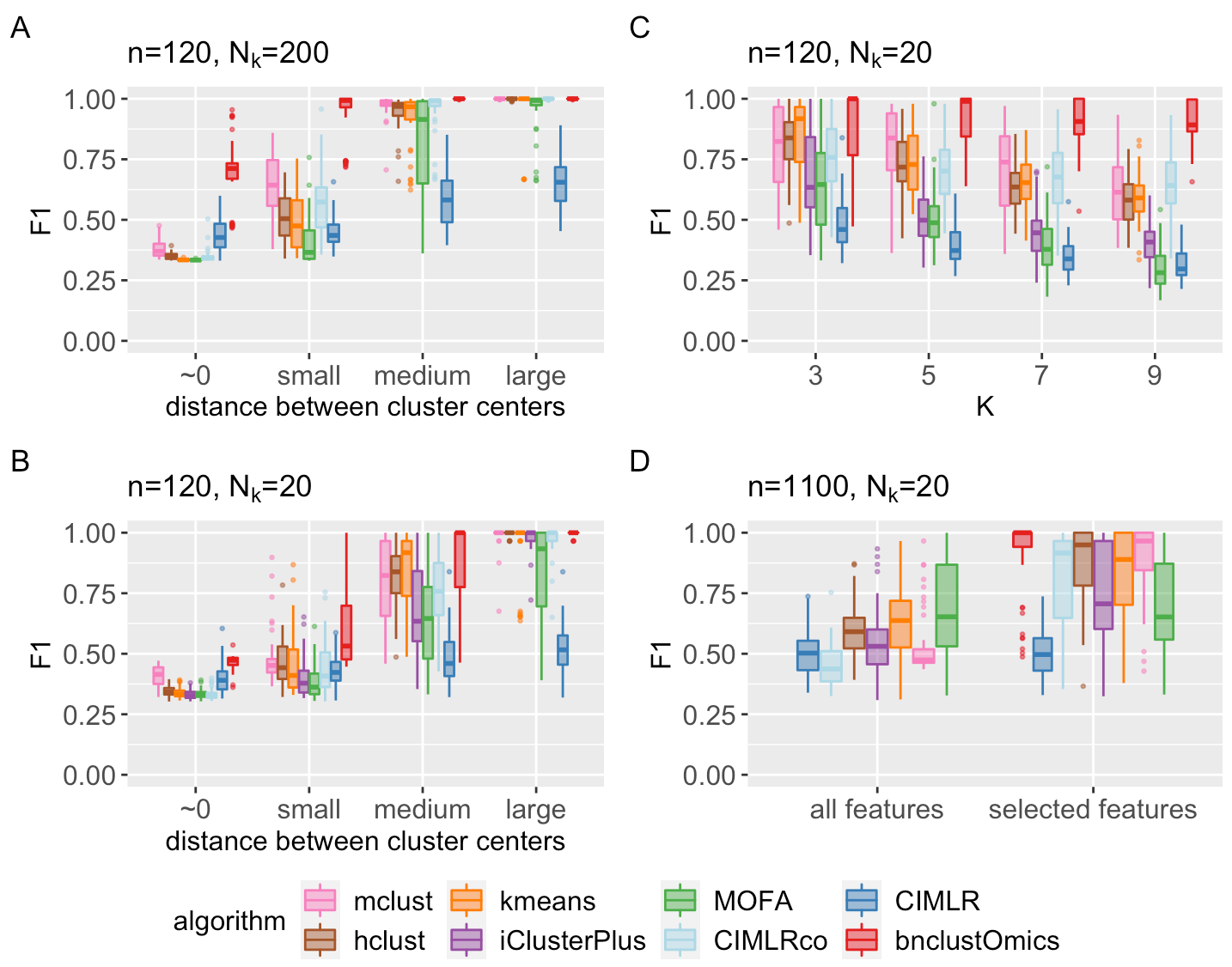

Supplement: S2 Fig — 50 Bayesian network mixtures were generated for each simulation setting. For general clustering approaches, the dimension was reduced by applying PCA and running clustering on the first 5 principal components. All integrative multi-omics approaches were applied to the original data unless specified otherwise. CIMLRco denotes clustering results of the application of CIMLR to a subset of data consisting of observations of only continuous variables. NZk denotes the number of observations in one cluster, K the number of clusters, nc number of continuous nodes, nb number of binary nodes in networks. (A) K = 3, nc = 100, nb = 20, NZk=200 (B) K = 3, nc = 100, nb = 20, NZk=20 (C) nc = 100, nb = 20, NZk=20, K ∈ {3, 5, 7, 9}; distance between centers set to medium (D) K = 3, nc = 1000, nb = 100, NZk=20, algorithms were applied to the full data and a subset of data consisting of all binary nodes with non-zero standard deviation and 150 selected continuous nodes; distance between centers set to medium. (PNG) [file pcbi.1009767.s002.png]

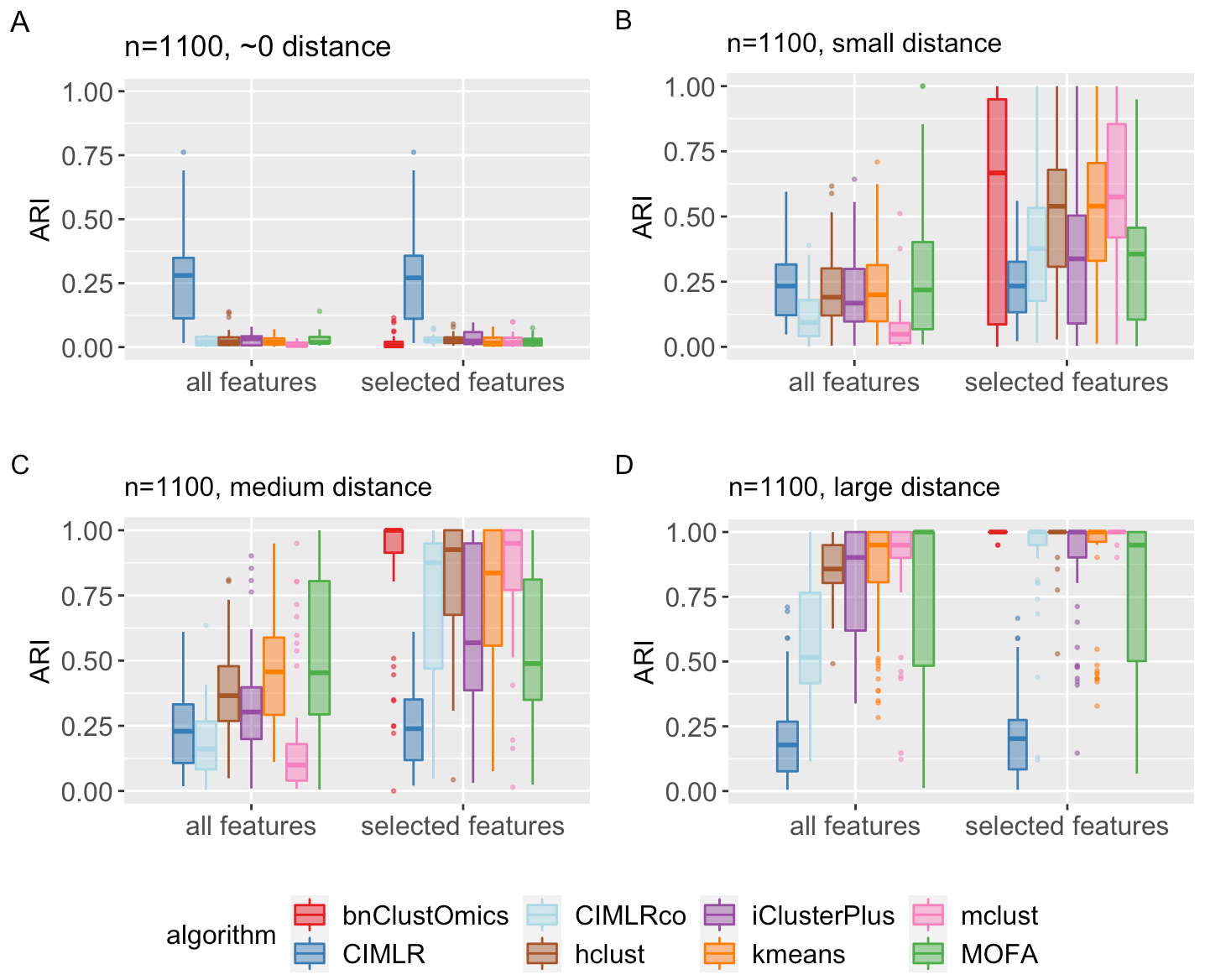

Supplement: S3 Fig — 50 Bayesian network mixtures were generated for each simulation setting. K = 3, nc = 1000, nb = 100, NZk=20, algorithms were applied to the full data and a subset of data consisting of all binary nodes with non-zero standard deviation and 150 selected continuous nodes; distance between centers set to medium. Distances between cluster centers are regulated by two parameters: SHD between networks in different clusters divided by the number of edges in one network (η) and the proportion of nodes with non-equal means between clusters (δ). (A) η = 0.1, δ = 0.00 (B) η = 0.2, δ = 0.03 (C) η = 0.2, δ = 0.04 (D) η = 0.3, δ = 0.05. (PNG) [file pcbi.1009767.s003.png]

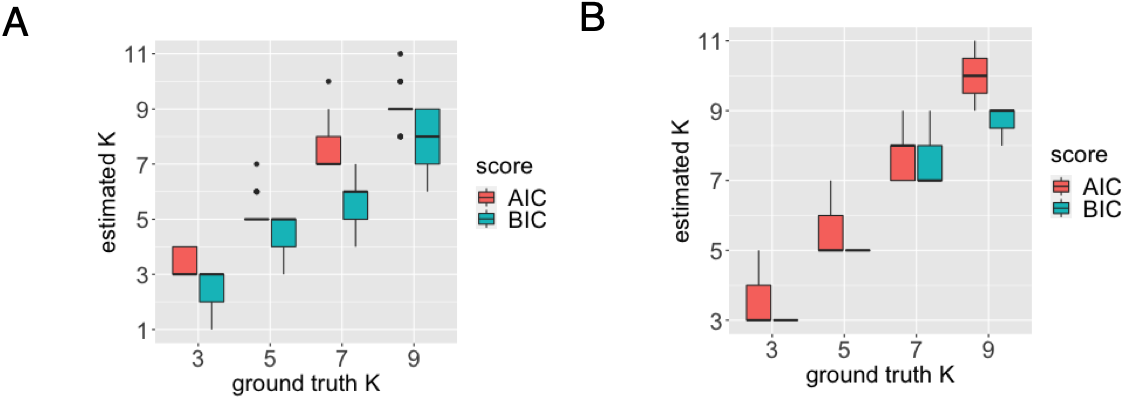

Supplement: S4 Fig — 30 Bayesian network mixtures were generated for each number of clusters K ∈ {3, 5, 7, 9} (ground truth). bnClustOmics was applied for each estimated K ∈ {1, …, 11} to each generated dataset and K^ was determined by minimizing the AIC or BIC score. The simulation was performed with two values for the number of observations (A) NZk=20 (B) NZk=200. (PNG) [file pcbi.1009767.s004.png]

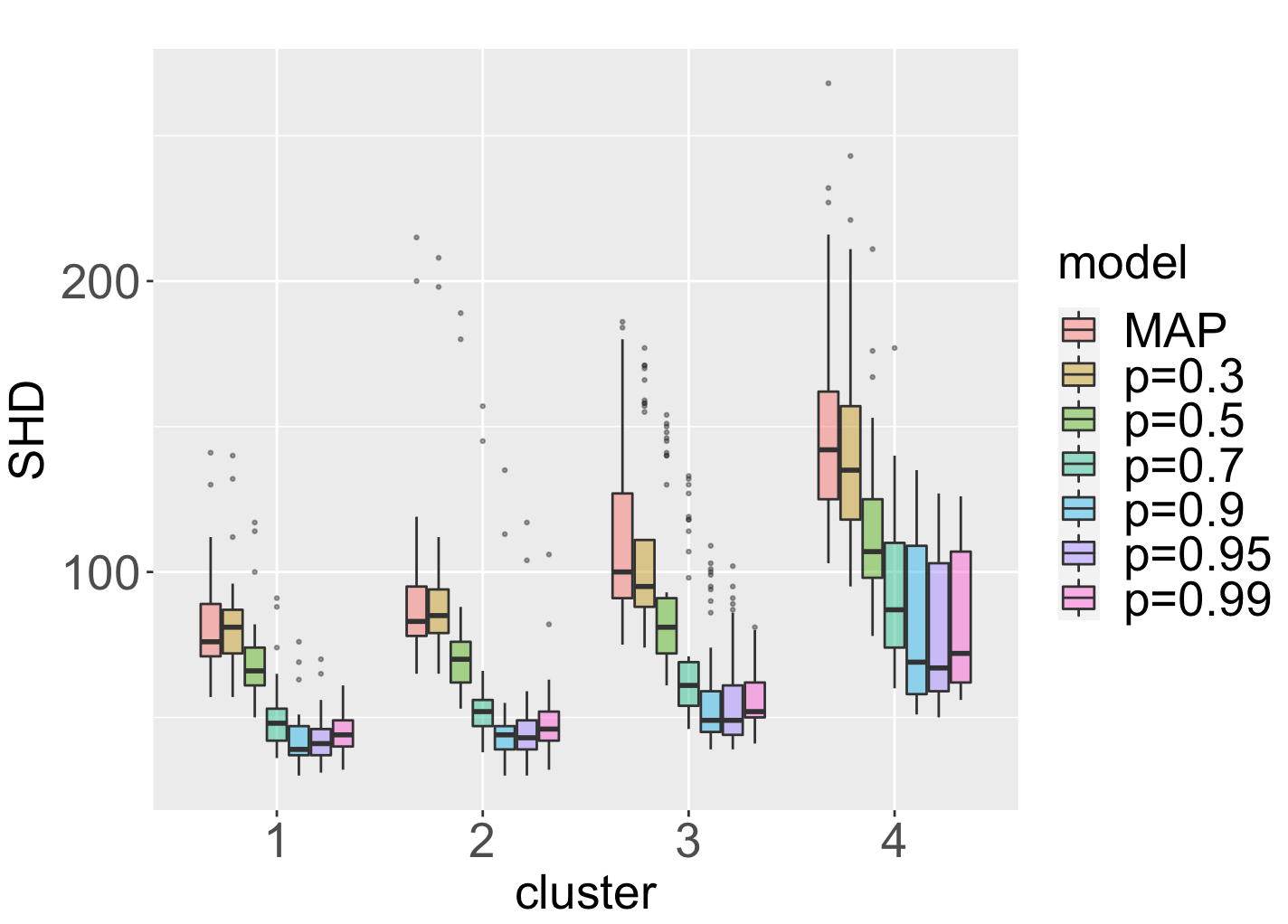

Supplement: S5 Fig — 50 BN mixtures were generated with unequal mixture weights: NZ1=150, NZ2=100, NZ3=50, NZ4=20 (cluster 1, cluster 2, cluster 3 and cluster 4). Distance between cluster centers is set to medium. bnClustOmics was used for clustering. The output MAP and consensus structures were compared to the ground truth CPDAG. (PNG) [file pcbi.1009767.s005.png]

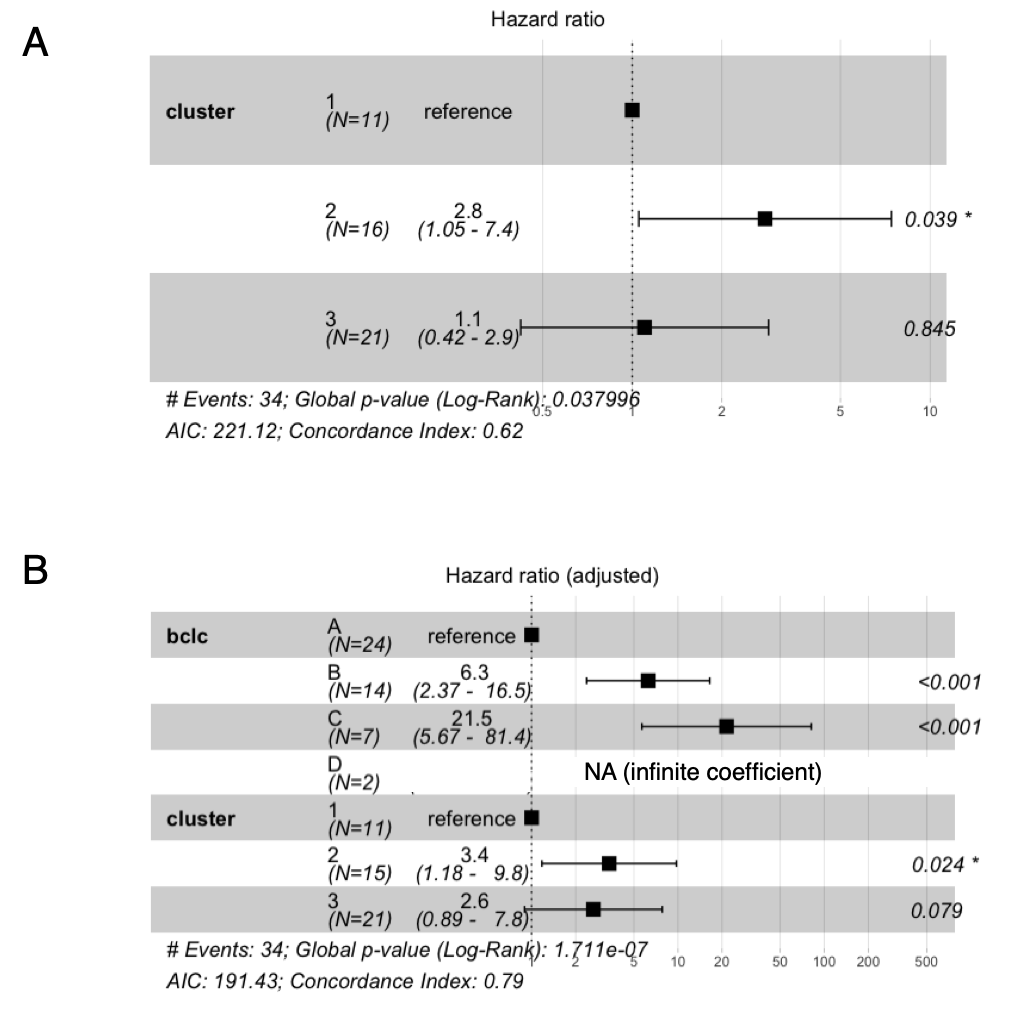

Supplement: S6 Fig — Hazard ratios of discovered clusters without (A) and with (B) adjustment for the BCLC stage. (PNG) [file pcbi.1009767.s006.png]

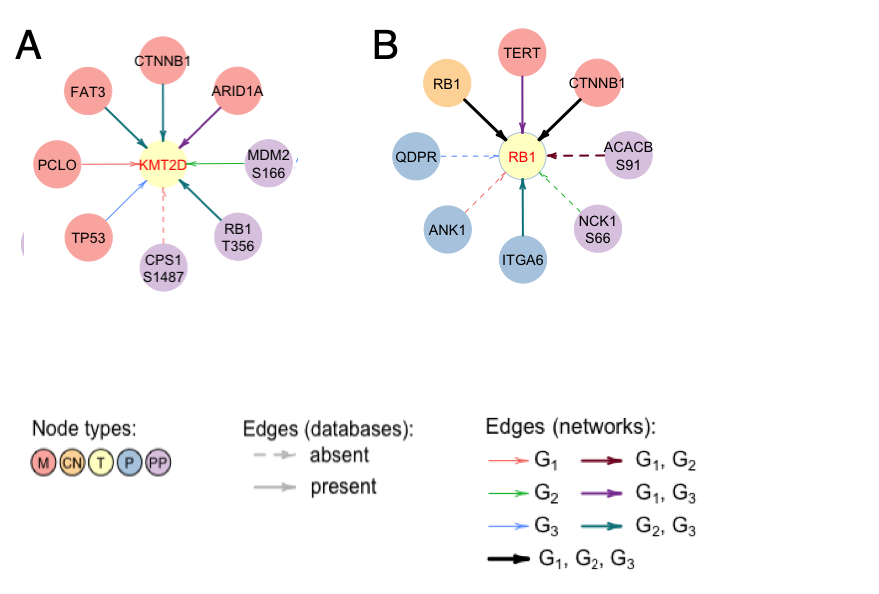

Supplement: S7 Fig — (A) connections of the node KMT2D-T (B) connections of the node RB1-T. (PNG) [file pcbi.1009767.s007.png]

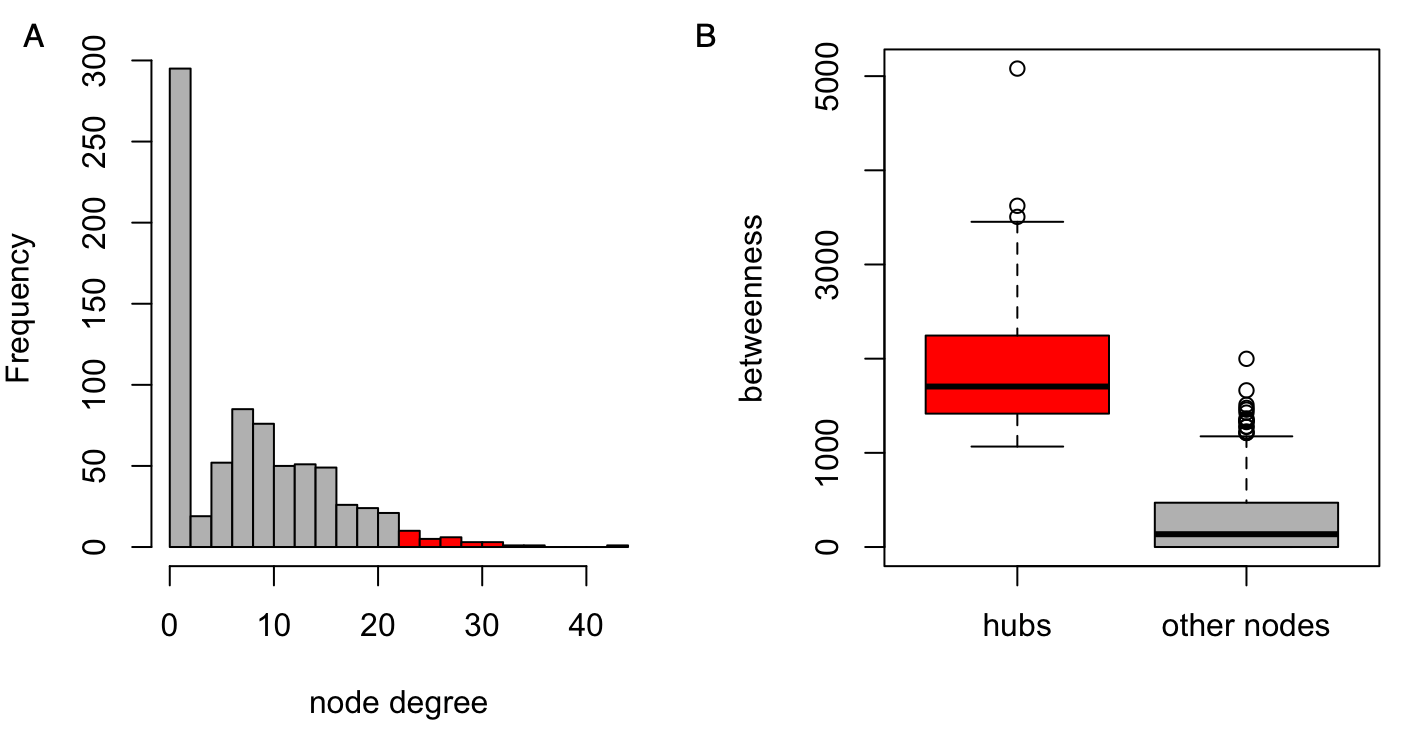

Supplement: S8 Fig — (A) Degree distribution of the network consisting of edges from all clusters for which one of the two requirements holds: the sum of posteriors of this edge in all clusters is grater than 1.2 or its posterior in one of the clusters is greater than 0.9. Red bars indicate nodes whose degree is over 20. (B) The betweenness of hub nodes and all other nodes. (PNG) [file pcbi.1009767.s008.png]
