# Supplementary material for: Multi-omics subtyping of hepatocellular carcinoma patients using a Bayesian network mixture model
Source: PLoS Comput Biol. 2022 Sep 6;18(9):e1009767. doi: 10.1371/journal.pcbi.1009767 (PMC9481159; doi:10.1371/journal.pcbi.1009767)
Supplement: S1 Appendix — (PDF) [file pcbi.1009767.s009.pdf]

S1 Appendix

The steps of generating a Bayesian network mixture and the dataset from this mixture include:

1. Generating DAGs G_k, $k = 1, ..., K$, consisting of n variables each, n_b binary and n_c continuous.

   - First, we generate a random DAG G_1 consisting of n_c continuous nodes with the function randomDAG from the R-package pcalg [1]. The parameter prob is set to $\frac{2}{n_c}$ and corresponds to each continuous node $X_{\psi k}$ having one continuous parent on average. Then, the remaining $K - 1$ DAGs in the mixture are generated such that the SHD between each of them and the first structure equals $\eta|E|$, where $|E|$ is the number of edges in the first randomly generated structure. We use η values in the range $0.1 - 0.4$, hence graphs representing different mixture components have a lot of edges in common. From a biological point of view, this makes sense: While some interactions may be altered in a particular cancer subtype, most of them will stay the same (e.g. housekeeping pathways).
   - At the next step, we add random edges from binary to continuous nodes, such that each binary node has 0.5 continuous children on average. These edges model the effects of mutations on other nodes (e.g. transcripts, proteins) and are generated randomly for each mixture component.

2. Generating parameters of local probability distributions (LPDs) for each structure.

   - For 99% of binary nodes $X_{\omega k}$, frequencies $\lambda_{\omega k}$ are sampled from beta distribution with parameters $\alpha = 0.1$, $\beta = 7$, modeling sparse and heterogeneous mutation data. For 1% of binary variables (minimum one variable) frequencies $\lambda_{\omega k}$ are sampled from a beta distribution with parameters $\alpha = 0.5$ and $\beta = 1$, modeling rare genes, for which higher frequencies are observed in known cancer subtypes.
   - For continuous nodes: regression coefficients $\beta_{\psi k}$ for nodes with non-empty parent sets are chosen in the range $[0.5, 1.5]$. Conditional standard deviations $\sigma_{\psi k}$ are sampled from a normal distribution with mean 0.3 and standard deviation 0.2; to prevent negative values, we use the absolute values of generated numbers.
   - Regression intercepts $m_{\psi k} = 0$, by default, apart $v = \delta n_c$ nodes G_k. For these v nodes, we first sample the *sign* ("+" or "-") with equal probability and then sample randomly in the range $[0.5, 1.5]$ or $[-1.5, -0.5]$. The parameter δ directly impacts how far the centers of distributions μ_k are from each other.

3. Generating data for each mixture component Z_k, $k = 1, ..., K$ using graphs and parameters generated in the previous steps.

- Generate N_{Z_k} observations of each binary node $X_{\omega k}$ from a Bernoulli distribution, using parameters $\lambda_{\omega k}$.
- Generate N_{Z_k} observations of each continuous node $X_{\psi k}$ according linear Gaussian model, using parameters G_k, m_k, B_k, σ_k.

We varied two parameters of generated Bayesian network mixtures to see how different algorithms performed depending on the signal strength, defined as $L2$ norm between centers of distributions of mixture components. The first parameter η is responsible for the structural difference between networks representing mixture components. The second parameter δ was responsible for differences between vectors of regression intercepts m_k, $k = 1, ..., K$. The tables below represent the correspondence between labels used to characterize the distances between cluster centers, parameters η, δ and average L_2 norm of differences between vectors of unconditional means μ_i and μ_j, $i \neq j$.

Table A: **Distances between cluster centers, $n = 120$.** Correspondence between parameters η, δ and labels used to define the distances between cluster centers ($n = 120$, $n_b = 20$, $n_c = 100$). The fourth column represents average L_2 norm between pairs of μ_i and μ_j, $i \neq j$ for all generated mixtures; the range is given in brackets.

Distance	η	δ	average L_2
no	0.1	0	0.27 (0.03, 0.85)
small	0.2	0.1	5.78 (4.24, 6.86)
medium	0.3	0.2	9.44 (7.89, 10.73)
large	0.4	0.4	14.28 (12.89, 17.37)

Table B: **Distances between cluster centers, $n = 1100$.** Correspondence between parameters η, δ and labels used to define the strength of the signal ($n = 1100$, $n_b = 100$, $n_c = 1000$). The fourth column represents average L_2 norm computed pairwise for all μ_i and μ_j, $i \neq j$ for all generated mixtures; the range is given in brackets.

Distance	η	δ	average L_2
no	0.1	0.00	2.15 (1.96, 2.36)
small	0.2	0.03	11.57 (10.74, 13.36)
medium	0.2	0.04	13.08 (11.92, 14.86)
large	0.3	0.05	19.45 (17.14, 22.35)

References

[1] Markus Kalisch et al. “Causal Inference Using Graphical Models with the R Package pcalg”. In: *Journal of Statistical Software* 47.11 (2012). DOI: 10.18637/jss.v047.i11. URL: https://doi.org/10.18637/jss.v047.i11.
