# Supplementary material for: Multi-omics subtyping of hepatocellular carcinoma patients using a Bayesian network mixture model
Source: PLoS Comput Biol. 2022 Sep 6;18(9):e1009767. doi: 10.1371/journal.pcbi.1009767 (PMC9481159; doi:10.1371/journal.pcbi.1009767)
Supplement: S2 Appendix — (PDF) [file pcbi.1009767.s010.pdf]

S2 Appendix

This simulation study illustrates clustering accuracy of bnClustOmics depending on the selection of relevant features. For simulations we use $n_c = 1000$ (similarly to the feature selection benchmarking study [1]) and $n_b = 100$. For clustering, we choose only binary features which equal to 1 in at least one generated sample. In addition we try several approaches to select the continuous features:

1. random selection: 150 continuous features
2. moCluster: approximately 150 continuous features with non-zero loadings defined by sparse consensus PCA (sparsity coefficient 0.06)
3. SGCCA [2]: approximately 150 continuous features (sparsity coefficient $c1 = 0.26$)
4. MOFA: top 150 features sorted by total absolute weight in all latent factors
5. ranking by the absolute value of normalized mean: top 150 features sorted by $|\frac{\overline{X}_\psi}{s_\psi}|$, where $\overline{X}_\psi$ is sample mean and s_ψ is sample standard deviation of X_ψ
6. hybrid of 4. and 5.: mix of MOFA (75 features) and ranking by the absolute value of normalized mean $|\frac{\overline{X}_\psi}{s_\psi}|$ (75 features).

By simulation study design, the default values for all continuous nodes are 0. Hence a ranking by the normalized mean $|\frac{\overline{X}_\psi}{s_\psi}|$ is similar to a ranking by the one-sample *t-test* statistics and defines the variables which most extremely deviate from 0. Feature selection using normalized mean can be seen as a proxy to performing the DGE analysis in the real expression data and selecting genes whose expression mostly deviates from non-tumor samples.

The method moCluster performed best with regard to feature selection in the benchmarking study [1]. However, in that study, the authors generated all variables independently and did not include any interactions in the model. In our simulation, this approach did not perform well. Ranking by normalized mean and hybrid approach has shown the best performance. However, the hybrid approach preserved more edges from the original network in subnetworks consisting of selected nodes only. For this reason, when comparing the accuracy of bnClustOmics to other methods in in the manuscript, we show the accuracy of bnClustOmics with the hybrid approach being used for feature selection.

Figure A: (A) Accuracy of bnClustOmics with different feature selection approaches. (B) The total number of edges from all generated networks which are also present in subnetworks consisting of selected nodes. (C) number of features selected by each approach.

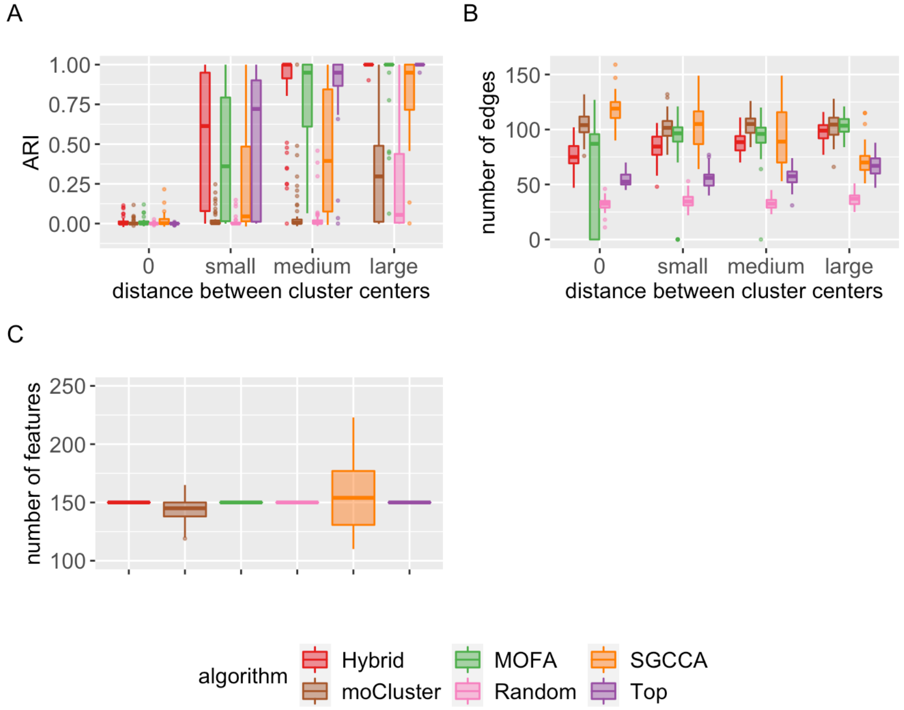

References

[1] Morgane Pierre-Jean et al. “Clustering and variable selection evaluation of 13 unsupervised methods for multi-omics data integration”. In: *Briefings in Bioinformatics* 21.6 (Dec. 2019), pp. 2011–2030. DOI: 10.1093/bib/bbz138. URL: https://doi.org/10.1093/bib/bbz138.

[2] A. Tenenhaus et al. “Variable selection for generalized canonical correlation analysis”. In: *Biostatistics* 15.3 (Feb. 2014), pp. 569–583. DOI: 10.1093/biostatistics/kxu001. URL: https://doi.org/10.1093/biostatistics/kxu001.
