# Supplementary material for: Multi-omics subtyping of hepatocellular carcinoma patients using a Bayesian network mixture model
Source: PLoS Comput Biol. 2022 Sep 6;18(9):e1009767. doi: 10.1371/journal.pcbi.1009767 (PMC9481159; doi:10.1371/journal.pcbi.1009767)
Supplement: S3 Appendix — (PDF) [file pcbi.1009767.s011.pdf]

S3 Appendix

To select M nodes, we included all mutated genes found significant by MutSigCV tool ($q < 0.1$, [3]). In addition, we have added the genes which were found significantly mutated in the TCGA HCC cohort, and the genes identified by HCC studies [5, 6, 2] as potential cancer drivers if they were mutated in at least two samples in the HCC dataset.

For nodes of continuous types, we first identified latent factors using MOFA on a subset of features passing standard deviation thresholds (1 for proteome and 2 for transcriptome and phosphoproteome). Five latent factors have been identified by MOFA. Consequently, we selected the top 50 features for each omics type by the total absolute weight of features in all latent factors.

We extended the selected P and PP features by performing the DGE analysis and picking the differentially expressed features (q <0.05), which are also present in the kinase-substrate database Omnipath. Their crucial role in cancer development explains our interest in kinases. Most protein kinases promote cell proliferation, survival, and migration. Furthermore, their aberrant activity is often associated with cancer development [1]. The standard-of-care HCC treatment, Sorafenib, is also a multi-kinase inhibitor.

For P nodes, we also selected differentially expressed features present in the transcription factor (TF) database Omnipath, confidence level B.

We have also extended each omics feature set with genes present in selected features of other omics sets for consistency and interpretability of networks. For example, for the possibility of discovering an edge *TP53-M* $\rightarrow$ *TP53-P*, we have included TP53 at the protein level. The same reasoning stands behind our choice of CN nodes, which were selected as a union of gene features selected from transcriptome, proteome, and phosphoproteome. In addition, we included CN nodes identified as potential drivers in [4]. We excluded CN nodes that had 0 variance in the HCC dataset.

Table A: **Summary of feature selection for each omics type.** The features are selected as a union of features satisfying the listed criteria. n_O denotes the number of selected features per each omics type.

omics type	criteria	n_O
M	MutSigCV (q<0.1)<br>Identified in [5, 6, 2]	24
CN	M, T, P and PP features<br>Identified in [4]	292
T	MOFA top 50 by absolute weight,<br>P features<br>Targets of P features chosen as TF	188
P	MOFA top 50 by absolute weight<br>M, T features<br>DE (q<0.05) + present in Omnipath TF database<br>DE (q<0.05) + present in Omnipath KS database	116
PP	MOFA top 50 by absolute weight<br>DE (q<0.05) + present in Omnipath KS database	158
all		778

References

[1] Khushwant S. Bhullar et al. "Kinase-targeted cancer therapies: progress, challenges and future directions". In: *Molecular Cancer* 17.1 (Feb. 2018). DOI: 10.1186/s12943-018-0804-2. URL: https://doi.org/10.1186/s12943-018-0804-2.

[2] Fanyun Kong et al. "Integrative analysis of highly mutated genes in hepatitis B virus-related hepatic carcinoma". In: *Cancer Medicine* 9.7 (Apr. 2020), pp. 2462–2479. DOI: 10.1002/cam4.2903. URL: https://doi.org/10.1002/cam4.2903.

[3] Michael S. Lawrence et al. "Mutational heterogeneity in cancer and the search for new cancer-associated genes". In: *Nature* 499.7457 (June 2013), pp. 214–218. DOI: 10.1038/nature12213. URL: https://doi.org/10.1038/nature12213.

[4] Charlotte K Y Ng et al. "Proteogenomic characterization of hepatocellular carcinoma". In: *bioRxiv* (Mar. 2021). DOI: 10.1101/2021.03.05.434147. URL: https://doi.org/10.1101/2021.03.05.434147.

[5] Chinthalapally V. Rao, Adam S. Asch, and Hiroshi Y. Yamada. "Frequently mutated genes/pathways and genomic instability as prevention targets in liver cancer". In: *Carcinogenesis* 38.1 (Nov. 2016), pp. 2–11. DOI: 10.1093/carcin/bgw118. URL: https://doi.org/10.1093/carcin/bgw118.

[6] Yuannv Zhang et al. "Integrated Analysis of Mutation Data from Various Sources Identifies Key Genes and Signaling Pathways in Hepatocellular Carcinoma". In: *PLoS ONE* 9.7 (July 2014). Ed. by Nathalie Wong,

e100854. DOI: 10.1371/journal.pone.0100854. URL: https://doi.org/10.1371/journal.pone.0100854.
