# Supplementary material for: Multi-omics subtyping of hepatocellular carcinoma patients using a Bayesian network mixture model
Source: PLoS Comput Biol. 2022 Sep 6;18(9):e1009767. doi: 10.1371/journal.pcbi.1009767 (PMC9481159; doi:10.1371/journal.pcbi.1009767)
Supplement: S4 Appendix — (PDF) [file pcbi.1009767.s012.pdf]

S4 Appendix

S4.1 Equivalent parametrizations of linear Gaussian Bayesian networks

In this subsection, we drop indices k, so the equations are valid for all mixture components. There are two ways to parametrize a Gaussian Bayesian network. One way is via a vector of regression intercepts m, a noise vector σ, and regression coefficients $B = \{\beta_\psi\}$ in the linear Gaussian model. The second way is via a vector of unconditional means μ and a covariance matrix Σ. Given the DAG G, the parameters $\{\mu, \Sigma\}$ can be transformed into equivalent parameters $\{m, B, \sigma\}$ [1], where regression coefficients β_ψ are computed only for those nodes, which have non-empty parent sets in a DAG G. Let Σ_{VW} be the block of the covariance matrix consisting only of rows with indices V and columns with indices W. And let W be the parents of node V in the graph G, then

$$
\begin{aligned}
m_V &= \mu_V - \Sigma_{VW}\Sigma_{WW}^{-1}\mu_W \\
\beta_V &= \Sigma_{WW}^{-1}\Sigma_{VW} \\
\sigma_V^2 &= \Sigma_{VV} - \Sigma_{VW}\Sigma_{WW}^{-1}\Sigma_{WV}.
\end{aligned}
$$

For convenience, we use both parametrizations interchangeably. For example, in defining parameters of simulation studies it is more convenient to use $\{m, B, \sigma\}$, while in the description of the EM algorithm we use $\{\mu, \Sigma\}$.

S4.2 Estimation of parameters of mixed Bayesian networks

Let $N_k = \sum_{i=1}^N \gamma_{ik}$. For binary (mutation) nodes, we follow [3] and parametrize local probability distributions as $P(X_{\omega k} = 1) = \lambda_{\omega k}$, with a beta prior on $\lambda_{\omega k}$ with hyperparameters $\alpha = \beta = \frac{1}{4}$. The posterior of λ follows a beta distribution as well, so we compute the MAP parameters for all binary nodes X_ω in the M step of the EM algorithm as follows:

$$
\hat{\lambda}_{\omega k} = \frac{\frac{1}{4} + \sum_{i=1}^N \gamma_{\omega k} D_{i\omega}}{\frac{1}{2} + N_k}
$$

For continuous and ordinal nodes we use the BGe score and assume a normal-inverse-Wishart prior on the parameters μ and Σ [1]:

$$
\mu \sim \mathcal{N}(\nu, a_\mu \Sigma)
$$

$$
\Sigma \sim \mathcal{W}^{-1}(a_w, U).
$$

The posterior is then also normal-inverse-Wishart and the MAP parameters are computed as follows:

$$
\hat{\mu}_k = \frac{N_k \overline{D}_{Nk} + a_\mu \nu}{a_\mu + N_k}
$$

$$
\hat{\Sigma}_k = \frac{U + S_{Nk} + \frac{a_\mu N_k}{a_\mu + N_k}(\nu - \overline{D}_{Nk})(\nu - \overline{D}_{Nk})'}{a_w + N - n - 1},
$$

where $\overline{D}_{Nk} = \frac{\sum_{i=1}^N \gamma_{ik} D_i}{N_k}$ and $S_{Nk} = \sum_{i=1}^N \gamma_{ik}(D_i - \overline{D}_{Nk})(D_i - \overline{D}_{Nk})'$. The values of the hyperparameters a_μ, a_w, the prior mean vector ν and the parametric matrix U by default are set as follows [2]:

$$
a_\mu = 1
$$

$$a_w = n + a_\mu + 1$$

$$\nu = \overline{0}$$

$$U = \mathbb{1}\frac{a_\mu(a_w - n - 1)}{a_\mu + 1} = \frac{\mathbb{1}}{2},$$

where $\mathbb{1}$ is the identity matrix, and $\overline{0}$ is a vector consisting of zeros.

When MAP graphs G_k and parameters $\hat{\mu}_k$, $\hat{\Sigma}_k$ are estimated, the estimates $(\hat{m}_k,\ \hat{B}_k,\ \hat{\sigma}_k)$ are computed according to S4.1.

S4.3 Computation of local likelihoods

For binary nodes, we compute likelihoods as

$$P(D_{i\omega} \mid \hat{\lambda}_{\omega k}) = \hat{\lambda}_{\omega k}^{D_{i\omega}}(1 - \hat{\lambda}_{\omega k})^{1-D_{i\omega}}$$

for all binary nodes.

For continuous nodes X_ψ, we compute likelihoods according to the linear Gaussian model:

$$P(D_{i\psi} \mid \mathbf{Pa}_{\psi k}, \hat{\mu}_k, \hat{\Sigma}_k) = \frac{1}{\sqrt{2\pi\hat{\sigma}^2_{\psi k}}} \exp\left(\frac{D_{i\psi} - \hat{m}_{\psi k} - \sum_{\xi : X_{\xi k} \in \mathbf{Pa}_{\psi k}} \hat{\beta}^{\xi}_{\psi k} D_{ij}}{2\hat{\sigma}^2_{\psi k}}\right)$$

For copy number nodes X_ϕ, we use a similar Gaussian likelihood, but the sum over parents is dropped due to structural assumptions (copy number nodes are not allowed to have incoming edges):

$$P(D_{i\phi} \mid \hat{\mu}_k, \hat{\Sigma}_k) = \frac{1}{\sqrt{2\pi\hat{\sigma}^2_{\phi k}}} \exp\left(\frac{D_{i\phi} - \hat{m}_{\phi k}}{2\hat{\sigma}^2_{\phi k}}\right)$$

.

References

[1] Dan Geiger and David Heckerman. "Parameter priors for directed acyclic graphical models and the characterization of several probability distributions". In: *The Annals of Statistics* 30.5 (Oct. 2002). DOI: 10.1214/aos/1035844981. URL: https://doi.org/10.1214/aos/1035844981.

[2] Marco Grzegorczyk. "An Introduction to Gaussian Bayesian Networks". In: *Systems Biology in Drug Discovery and Development.* Humana Press, 2010, pp. 121–147. DOI: 10.1007/978-1-60761-800-3_6. URL: https://doi.org/10.1007/978-1-60761-800-3_6.

[3] Jack Kuipers et al. "Mutational interactions define novel cancer subgroups". In: *Nature Communications* 9.1 (Oct. 2018). DOI: 10.1038/s41467-018-06867-x. URL: https://doi.org/10.1038/s41467-018-06867-x.
