# Supplementary material for: Multi-omics subtyping of hepatocellular carcinoma patients using a Bayesian network mixture model
Source: PLoS Comput Biol. 2022 Sep 6;18(9):e1009767. doi: 10.1371/journal.pcbi.1009767 (PMC9481159; doi:10.1371/journal.pcbi.1009767)
Supplement: S5 Appendix — (PDF) [file pcbi.1009767.s013.pdf]

S5 Appendix

For transcriptome data, the pre-processing steps included:

- Like in [2] gene-level expected counts were upper-quartile-normalized to 1000.
- $\log_2$ transformation.

For proteome data, the pre-processing steps included:

- $\log_2$ transformation.
- Normalization by median substraction.
- Filtering out proteins which were detected in less than 50% of samples.
- For clustering only: imputation of missing values using the R package impute [3]. For differential expression analysis, we used unimputed values.

For phosphoproteome data, the pre-processing steps included:

- $\log_2$ transformation.
- Normalization by median substraction.
- Filtering out proteins which were detected in less than 50% of samples.
- Batch correction with the R package edgeR.
- For clustering only: imputation of missing values using the R package impute [3]. For differential expression analysis, we used unimputed values.

The CNA data was obtained at the gene level from the study by Ng et al. [2]. The copy number status was derived from the log-ratio and takes values from 2 to -2, which denote [1]:

- 2: amplification
- 1: copy gain indicates a low-level gain
- 0: copy number neutral
- -1: shallow deletion, indicating a heterozygous deletion
- -2: deep deletion, indicating a homozygous deletion

This way, despite the ordinal nature of the CNA data, the range of the values justifies the normal approximation.

References

[1] Ethan Cerami et al. "The cBio Cancer Genomics Portal: An Open Platform for Exploring Multidimensional Cancer Genomics Data." In: *Cancer Discovery* 2.5 (May 2012), pp. 401–404. DOI: 10.1158/2159-8290.cd-12-0095. URL: https://doi.org/10.1158/2159-8290.cd-12-0095.

[2] Charlotte K Y Ng et al. "Proteogenomic characterization of hepatocellular carcinoma". In: *bioRxiv* (Mar. 2021). DOI: 10.1101/2021.03.05.434147. URL: https://doi.org/10.1101/2021.03.05.434147.

[3] Robert Tibshirani Trevor Hastie. *impute*. 2017. DOI: 10.18129/B9.BIOC.IMPUTE. URL: https://bioconductor.org/packages/impute.
