# Supplementary material for: Multi-omics subtyping of hepatocellular carcinoma patients using a Bayesian network mixture model
Source: PLoS Comput Biol. 2022 Sep 6;18(9):e1009767. doi: 10.1371/journal.pcbi.1009767 (PMC9481159; doi:10.1371/journal.pcbi.1009767)

S6 Appendix

We investigated the edges outgoing from *TP53-M* whose posterior probabilities in G_2 is higher than 0.9 (p_cl2> 0.9, Table A). All four identified edges are specific to G_2, as their posteriors in other networks are lower than 0.4. We further considered only gene products that were differentially expressed in cluster 2 (p_DE2< 0.05). Three edges satisfied these criteria. Eventually, we focussed on the edge connecting *TP53-M* and *TERT-T*, since it was also found in the STRING database.

Table A. Connections of *TP53-M* node whose posterior probability is larger than 0.9 in network G_2 representing cluster 2. p_cl columns report posterior probabilities for corresponding clusters; p_DE columns report adjusted *p*-values of nodes in column "to" from the DGE analysis.

from	to	type1	type2	gene1	gene2	database	p_cl1	p_cl2	p_cl3	p_DE1	p_DE2	p_DE3
TP53	ENSG00000164362	M	T	TP53	TERT	TRUE	0.39	0.92	0.18	< 0.01	< 0.01	< 0.01
TP53	Q9BWD1	M	P	TP53	ACAT2	FALSE	0.00	0.95	0.00	0.31	< 0.01	< 0.01
TP53	Q13435_S436	M	PP	TP53	SF3B2	TRUE	0.39	0.93	0.17	0.76	0.95	0.77
TP53	Q9BW71_S160	M	PP	TP53	HIRIP3	FALSE	0.00	0.98	0.21	< 0.01	0.01	0.02

Fig A. *TP53-M* node and its neighbors in networks representing three clusters identified by bnClustOmics.

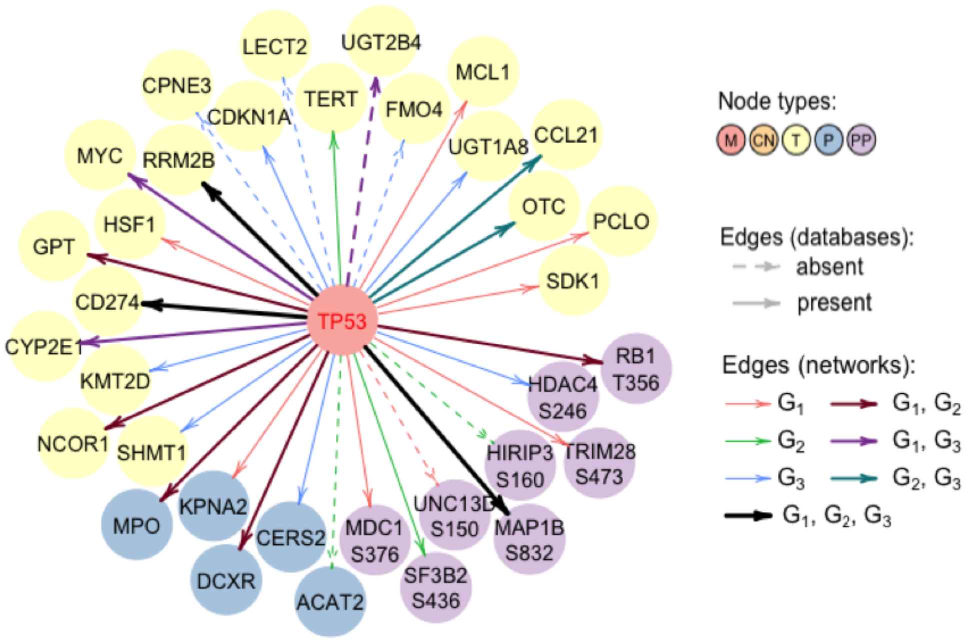

Fig B. Log2-fold changes between expression of *TERT-T* in three HCC clusters and mean expression of *TERT-T* in 15 healthy livers.

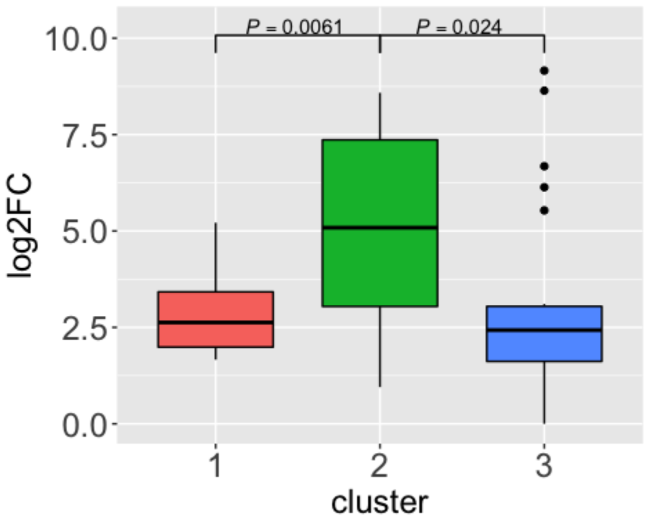

Supplement: S6 Appendix — Fig A. TP53- node and its neighbors in networks representing three clusters identified by bnClustOmics. Fig B. Log2-fold changes between expression of TERT- in three HCC clusters and mean expression of TERT- in 15 healthy livers. (PDF) [file pcbi.1009767.s014.pdf]
