# Supplementary material for: Multi-omics subtyping of hepatocellular carcinoma patients using a Bayesian network mixture model
Source: PLoS Comput Biol. 2022 Sep 6;18(9):e1009767. doi: 10.1371/journal.pcbi.1009767 (PMC9481159; doi:10.1371/journal.pcbi.1009767)
Supplement: S7 Appendix — (PDF) [file pcbi.1009767.s015.pdf]

S7 Appendix

Six out of all 48 patients in the analyzed cohort were treated with Sorafenib. All of these patients were assigned to either cluster 2 or cluster 3 and none to cluster 1. Edmondson grade, survival, and side-effects to Sorafenib were mostly similar within clusters. Two of three patients in cluster 2 experienced hepatic decompensation within several days after the start of treatment and had to stop it. Patients from cluster 3 could, on average, tolerate the side effects longer. All patients in cluster 3 survived longer than patients in cluster 2 even within the same clinical stage.

Table A. Clinical information about patients who received treatment with Sorafenib and ware assigned to cluster 2 in the clustering by bnClustOmics. Survival time and length of treatment are reported in days.

sample ID	Edmondson	age_diagnosis	death	survival time	BCLC	treatment length	reason to stop
C177c	3	18	1	45	C	8	Hepatic Decompensation
C383b	3	48	1	94	B	6	Hepatic Decompensation
B763b	4	60	1	385	A	123	

Table B. Clinical information about patients who received treatment with Sorafenib and were assigned to cluster 3 in the clustering by bnClustOmics. Survival time and length of treatment are reported in days.

sample ID	Edmondson	age_diagnosis	death	survival time	BCLC	treatment length	reason to stop
C795	2	53	1	625	A	58	Intolerant to side-effects
C346b	2	72	0	1663	A	118	Intolerant to side-effects
B983b	2	74	1	485	B	57	Intolerant to side-effects
