# Supplementary material for: Multi-omics subtyping of hepatocellular carcinoma patients using a Bayesian network mixture model
Source: PLoS Comput Biol. 2022 Sep 6;18(9):e1009767. doi: 10.1371/journal.pcbi.1009767 (PMC9481159; doi:10.1371/journal.pcbi.1009767)
Supplement: S1 Table — Summary of the likelihood ratio test for Cox proportional hazards models based on assignments obtained by clustering algorithms. The models were fitted for k = 3 as found optimal by bnClustOmics or for other k that was found optimal by method-specific tools or the elbow method when no such tool was available. For all algorithms apart from bnClustOmics and MOFA, all available omics features were used as input. For MOFA, standard deviations filters (1 for P features, 2 for T and PP, 0.5 for CN features) were applied as recommended by the authors of the method. Models with the number of clusters found by each model-specific method are marked with * in the column ‘best’. (PDF) [file pcbi.1009767.s016.pdf]

S1 Table

algorithm	k	best	p-value	p-value (BCLC-adjusted)
mclust	3	*	0.17	0.32
hclust	3		0.002	0.137
hclust	6	*	0.02	0.26
kmeans	3		0.002	0.77
kmeans	6	*	0.86	0.14
iClusterPlus	3		0.052	0.51
iClusterPlus	2	*	0.98	0.38
CIMLR	3	*	0.94	0.39
CIMLR (T,P,PP)	3	*	0.017	0.20
MOFA	3		0.15	0.25
MOFA	6	*	0.56	0.47
bnClustOmics	3	*	0.038	0.043
