# Supplementary material for: Multi-omics subtyping of hepatocellular carcinoma patients using a Bayesian network mixture model
Source: PLoS Comput Biol. 2022 Sep 6;18(9):e1009767. doi: 10.1371/journal.pcbi.1009767 (PMC9481159; doi:10.1371/journal.pcbi.1009767)
Supplement: S2 Table — FDR values reflect the enrichment of KEGG signaling pathways with children of M nodes in cluster-specific networks. FDR values below 0.05 suggest significant enrichment. (PDF) [file pcbi.1009767.s017.pdf]

S2 Table

pathway	G_1	G_2	G_3
Hepatocellular carcinoma	0.0007	<0.0001	<0.0001
Proteoglycans in cancer	0.0015	0.0007	<0.0001
PI3K-Akt signaling	0.0002	0.0024	<0.0001
Cellular senescence	0.0185	0.0039	0.00051
wnt signaling	0.0033	>0.05	0.00058
p53 signaling	0.0219	0.0108	0.0079
Insulin signaling	>0.05	0.0109	<0.0001
mTOR signaling	>0.05	0.0157	0.0031
Cell Cycle	0.0095	0.0387	0.0071
AMPK signaling	>0.05	0.0387	0.0071
HIF-1 signaling	>0.05	>0.05	0.00062
MAPK signaling	>0.05	>0.05	0.0358
JAK-STAT signaling	>0.05	>0.05	0.0166
Rap-1 signaling	>0.05	>0.05	0.0358
Hippo signaling	0.0033	>0.05	>0.05
PD-L1 expression and PD-1 checkpoint pathway in cancer	>0.05	0.016	>0.05
Erbb signaling	>0.05	>0.05	<0.0001
