# Supplementary material for: Multi-omics subtyping of hepatocellular carcinoma patients using a Bayesian network mixture model
Source: PLoS Comput Biol. 2022 Sep 6;18(9):e1009767. doi: 10.1371/journal.pcbi.1009767 (PMC9481159; doi:10.1371/journal.pcbi.1009767)
Supplement: S3 Table — A list of phosphorylation sites with more than 15 cluster-specific interaction partners and their known functions in HCC and other cancers according to the PhosphoSitePlus database. (PDF) [file pcbi.1009767.s018.pdf]

S3 Table

ID	Gene_ID	DB	In liver studies	In HCC studies	Downstream effects	Upstream regulators	Known treatments
Q96T23_S1345	RSF1	yes	yes	-	-	-	yes
O15084_S1011	ANKRD28	yes	-	-	intracellular localization	CAMK2D	-
Q9UJM3_S273	ERRFI1	yes	yes	-	-	EGFR	-
Q9Y2T1_S70	AXIN2	yes	-	-	-	-	-
B1AK53_S642	ESPN	-	-	-	-	-	-
P00533_S1166	EGFR	yes	yes	yes	activates signalling cascades	CAMK2A	yes
P28482_T185	MAPK1	yes	yes	yes	altered apoptosis cell growth	-	yes
P05556_S785	ITGB1	yes	-	yes	cytoskeletal reorganization	PKCA	yes
P21333_S1734	FLNA	yes	-	-	-	-	-
O14828_S76	SCAMP3	yes	-	yes	-	-	yes
O60716_S252	CTNND1	yes	yes	yes	carcinogenesis induced	-	yes
P18031_S352	PTPN1	yes	-	-	-	-	-
P19878_S312	NCF2	yes	-	-	-	-	-
P21731_S331	TBXA2R	yes	-	-	signalling pathway regulation	PKACA	yes
P54646_S491	PRKAA2	yes	-	-	altered autophagy	-	yes
P62753_S244	RPS6	yes	-	yes	-	mTOR TSC2	yes
Q13557_T287	CAMK2D	yes	yes	yes	-	-	yes
Q14449_S372	GRB14	yes	-	yes	cell cycle regulation	-	yes
Q8IXS6_S228	PALM2	yes	-	-	-	-	-
