# Supplementary material for: Multi-omics subtyping of hepatocellular carcinoma patients using a Bayesian network mixture model
Source: PLoS Comput Biol. 2022 Sep 6;18(9):e1009767. doi: 10.1371/journal.pcbi.1009767 (PMC9481159; doi:10.1371/journal.pcbi.1009767)
Supplement: S4 Table — Allowed edges (i.e., not blacklisted) between and within omics types in the HCC analysis. Let X and Y denote gene names. Then, all edges from CN nodes to P nodes of the same genes are encoded as from X-CN to X-P. Edges between any two genes are encoded as edges between X-CN and Y-P (this includes the case when X equals Y). (PDF) [file pcbi.1009767.s019.pdf]

S4 Table

from	to	example of biological interpretation
X-M	Y-T, Y-P, Y-PP	functional interaction
X-CN	X-T, X-P, X-PP	central dogma of molecular biology
X-T	X-P, X-PP	central dogma of molecular biology
X-P	Y-T	transcription factor and its target, functional interaction
X-PP	Y-T	transcription factor and its target, functional interaction
X-P, X-PP	Y-P, Y-PP	physical interaction, functional interaction

The biological interpretation of most edges is straightforward, however, edges of type X-$M \rightarrow$ Y-$P/T/PP$, for two genes X and Y, are rarely considered when learning the networks. Mehnert et al. [1] have shown via experiments that a cancerous mutation in gene X can change the interactome of its protein product X-P without affecting the expression of X-P itself. Say Y-P is one of the interactors of X-P affected by a mutation in gene X. In this case, we can observe a statistical dependency between a mutation node X-M and a protein node Y-P (but not between X-P and Y-P). Such dependencies are particularly interesting because they help understand the links between genotypes and phenotypes.

References

[1] Martin Mehnert et al. "Multi-layered proteomic analyses decode compositional and functional effects of cancer mutations on kinase complexes". In: *Nature Communications* 11.1 (July 2020). DOI: 10.1038/s41467-020-17387-y. URL: https://doi.org/10.1038/s41467-020-17387-y.
