# Supplementary material for: Multi-omics subtyping of hepatocellular carcinoma patients using a Bayesian network mixture model
Source: PLoS Comput Biol. 2022 Sep 6;18(9):e1009767. doi: 10.1371/journal.pcbi.1009767 (PMC9481159; doi:10.1371/journal.pcbi.1009767)
Supplement: S5 Table — The network consists of edges from all clusters for which one of the two requirements holds: the sum of posteriors of this edge in all clusters is grater than 1.2 or its posterior in one of the clusters is greater than 0.9. The modules were identified by the function cluster_edge_betweenness from the package igraph [77]. (PDF) [file pcbi.1009767.s020.pdf]

S5 Table

Module	# Features	# Unique genes	KEGG pathways
63 PP, 16 T, 16 P	95	92	Focal Adhesion
10 PP, 5 T, 4 P	19	16	Insulin Signaling
15 PP, 1 T, 1 P	17	16	Pathways in Cancer, ERBB signaling
5 P, 2 T, 1 PP	8	6	Metabolic pathways
